# Quantitative proteomics of differentiated primary bronchial epithelial cells from chronic obstructive pulmonary disease and control identifies potential novel host factors post-influenza A virus infection

**DOI:** 10.3389/fmicb.2022.957830

**Published:** 2023-01-11

**Authors:** Misako Nakayama, Hannah Marchi, Anna M. Dmitrieva, Ashesh Chakraborty, Juliane Merl-Pham, Elisabeth Hennen, Ronan Le Gleut, Clemens Ruppert, Andreas Guenther, Kathrin Kahnert, Jürgen Behr, Anne Hilgendorff, Stefanie M. Hauck, Heiko Adler, Claudia A. Staab-Weijnitz

**Affiliations:** ^1^Institute of Lung Health and Immunity and Comprehensive Pneumology Center with the CPC-M BioArchive, Helmholtz Zentrum München, Member of the German Center of Lung Research (DZL), Munich, Germany; ^2^Division of Pathogenesis and Disease Regulation, Department of Pathology, Shiga University of Medical Science, Otsu, Japan; ^3^Core Facility Statistical Consulting, Helmholtz Zentrum München, Munich, Germany; ^4^Faculty of Business Administration and Economics, Bielefeld University, Bielefeld, Germany; ^5^Research Unit Lung Repair and Regeneration, Helmholtz Zentrum München, Member of the German Center of Lung Research (DZL), Munich, Germany; ^6^Metabolomics and Proteomics Core, Helmholtz Zentrum München, Neuherberg, Germany; ^7^Department of Internal Medicine, Medizinische Klinik II, Member of the German Center of Lung Research (DZL), Giessen, Germany; ^8^Department of Medicine V, Ludwig Maximilian University (LMU) Munich, Member of the German Center of Lung Research, University Hospital, Munich, Germany; ^9^Institute of Asthma and Allergy Prevention, Helmholtz Zentrum München, Member of the German Center of Lung Research (DZL), Munich, Germany

**Keywords:** organotypic, proteomics, flu receptor, primary human bronchial epithelial cells, COPD

## Abstract

**Background:**

Chronic obstructive pulmonary disease (COPD) collectively refers to chronic and progressive lung diseases that cause irreversible limitations in airflow. Patients with COPD are at high risk for severe respiratory symptoms upon influenza virus infection. Airway epithelial cells provide the first-line antiviral defense, but whether or not their susceptibility and response to influenza virus infection changes in COPD have not been elucidated. Therefore, this study aimed to compare the susceptibility of COPD- and control-derived airway epithelium to the influenza virus and assess protein changes during influenza virus infection by quantitative proteomics.

**Materials and methods:**

The presence of human- and avian-type influenza A virus receptor was assessed in control and COPD lung sections as well as in fully differentiated primary human bronchial epithelial cells (phBECs) by lectin- or antibody-based histochemical staining. PhBECs were from COPD lungs, including cells from moderate- and severe-stage diseases, and from age-, sex-, smoking, and history-matched control lung specimens. Protein profiles pre- and post-influenza virus infection *in vitro* were directly compared using quantitative proteomics, and selected findings were validated by qRT-PCR and immunoblotting.

**Results:**

The human-type influenza receptor was more abundant in human airways than the avian-type influenza receptor, a property that was retained *in vitro* when differentiating phBECs at the air–liquid interface. Proteomics of phBECs pre- and post-influenza A virus infection with A/Puerto Rico/8/34 (PR8) revealed no significant differences between COPD and control phBECs in terms of flu receptor expression, cell type composition, virus replication, or protein profile pre- and post-infection. Independent of health state, a robust antiviral response to influenza virus infection was observed, as well as upregulation of several novel influenza virus-regulated proteins, including PLSCR1, HLA-F, CMTR1, DTX3L, and SHFL.

**Conclusion:**

COPD- and control-derived phBECs did not differ in cell type composition, susceptibility to influenza virus infection, and proteomes pre- and post-infection. Finally, we identified novel influenza A virus-regulated proteins in bronchial epithelial cells that might serve as potential targets to modulate the pathogenicity of infection and acute exacerbations.

## 1. Introduction

Chronic obstructive pulmonary disease (COPD) is an umbrella term used to describe chronic and progressive lung diseases that cause limitations in lung airflow, which are not fully reversible. Currently, at least sixty-five million people suffer from moderate-to-severe COPD, with the prevalence being increasing and thus COPD has become the third leading cause of death worldwide (World Health Organization [WHO], 2021). The primary cause of COPD is tobacco smoke in high- and middle-income countries and biomass smoke and other fuels in low-income countries (Decramer et al., 2012). Exacerbation, defined as a sudden worsening of clinical manifestations and decrease in lung function, is related to a significantly worse survival outcome (Viniol and Vogelmeier, 2018), and the influenza virus is one of the respiratory viruses triggering COPD exacerbations (Bouquet et al., 2020). However, the underlying mechanisms of acute COPD exacerbations by influenza viruses have not been fully elucidated (Decramer et al., 2012; Viniol and Vogelmeier, 2018; Ji et al., 2022; Wu et al., 2022).

Respiratory viruses are associated with 40–64% of acute COPD exacerbations, and influenza virus infections cause substantial declines in lung function parameters (Smith et al., 1980). Seasonal influenza virus causes annual epidemics, with an estimation of 10% of the global population catching the disease, and the detection rates of influenza virus among hospitalized patients with acute exacerbation of COPD were reported as 8–36% (Mallia and Johnston, 2007; Viniol and Vogelmeier, 2018). Therefore, it is important to elucidate the mechanisms underlying the susceptibility and pathogenicity of influenza viruses in patients with COPD.

Given that the airway epithelium provides the first-line antiviral defense, it is a highly relevant question whether or not susceptibility to virus infection and virus replication is altered in COPD airway cells. Different responses of airway epithelium upon infection have been reported in patients with COPD compared with healthy controls (Hsu et al., 2015, 2016), suggesting that targeted treatment of exacerbations might serve to improve the prognosis of COPD.

Primary human bronchial epithelial cells (phBECs) differentiated at the air–liquid interface (ALI) represent a powerful tool to assess the airway-specific first-line defense against external stimuli. The resulting organotypic bronchoepithelium closely resembles the *in vivo* airway and is frequently used in studies of toxicology, infection, and drug delivery (Pezzulo et al., 2011; Lenz et al., 2014; Singanayagam et al., 2019; Berthold et al., 2022; Mastalerz et al., 2022). The fully differentiated epithelium, obtained 4 weeks after the airlift, contains all major bronchial epithelial cell types and displays physiologically important functional properties like mucus secretion and ciliary beating (Lodes et al., 2020). The model also allows for differentiating phBECs from different disease origins and assessing viral replication and antiviral epithelial responses after infection (Zarcone et al., 2017; Singanayagam et al., 2019).

Sialic acids on the surface of airway epithelial cells serve as receptors for influenza A viruses. Human influenza A viruses preferentially bind to sialic acids bound to galactose by the α2,6 linkage (SAα2,6Gal), the human-type flu receptor, whereas avian influenza A viruses preferentially bind to SAα2,3Gal, the avian-type flu receptor (Ito et al., 1997). Although the human airway epithelium predominantly displays the human-type flu receptor, the avian-type flu receptor has also been reported to be displayed by a minor proportion of cells (Shinya et al., 2006; Davis et al., 2015; Hsu et al., 2016). Considering that a major natural reservoir of influenza A virus is wild aquatic birds and that several subtypes of avian influenza A viruses have crossed the species barrier resulting in higher mortality rates than those caused by human influenza A viruses (Krammer et al., 2018), not only the human-type but also the avian-type flu receptor was assessed in this study.

The aims of this study were 2-fold. (1) To assess the susceptibility of airway epithelium of patients with COPD and controls to influenza virus. For this aim, we evaluated the expression of flu receptors in COPD and control lung sections as well as in a human organotypic model of the bronchial epithelium derived from COPD and control lung airway cells. (2) To identify novel molecular targets for the treatment of acute exacerbations caused by the influenza virus. Here, we performed quantitative proteomics in organotypic bronchoepithelia with phBECs derived from patients with COPD and controls.

## 2. Materials and methods

### 2.1. Subjects

Lectin-based and immunofluorescent staining of lung tissue was carried out in samples obtained from the UGMLC/DZL biobank, where biospecimen collection was approved by votes from the Ethics Committee of the Justus-Liebig-University School of Medicine (no. 111/08 and 58/15). Here, lung sections were from lung explants of patients with COPD, i.e., lungs with end-stage disease (GOLD stage IV; mean age 55 ± 6 years; three females, two males), or from donor’s lungs (mean age 52 ± 13 years, three females, two males).

All phBECs used in this study were obtained from the CPC-M BioArchive. PhBECs were derived from patients with COPD and controls, and were selected based on the following criteria: (1) male, (2) age over 60 years, (3) ex-smoker (more than 5 years), (4) a smoking history of more than 11 pack-years, and (5) the definition and classification provided by the Global Initiative for Chronic Obstructive Lung Disease (GOLD). The majority of phBECs, including all controls and COPD-derived phBECS from GOLD stage II/III, were derived from patients undergoing lung tumor resections and isolated from histologically normal regions adjacent to the resected lung tumors as assessed by an experienced pathologist. For the patients with COPD (*n* = 4), one patient was from GOLD stage IV, another from GOLD stage III, and two patients from GOLD stage II. The patient with COPD stage IV was a recipient of a lung transplant who had his lung resected at the University Hospital of the Ludwig Maximilians University Munich. Other patients underwent lobectomy at Asklepios clinic, Munich-Gauting, with a lung tumor as the primary diagnosis. All control subjects (*n* = 3) had normal lung functions and also underwent lobectomy at Asklepios clinic with a lung tumor as the primary diagnosis (Table 1). These patients received different multiple medications (outlined in Supplementary Table 1). The study was approved by the Ethics Committee of the Ludwig Maximilians University Munich, Germany (Ethic vote #333-10, #382-10). Written informed consent was obtained from all study participants.

**TABLE 1:** Patient information and bronchial diameter of specimens used for cell isolation.

	GOLD stage	Smoking status	Smoking-free period (years)	Pack-years	Sex	Age	Primary diagnosis	Bronchial diameter
COPD	IV	Ex	5	45	m	67	COPD	≈15 mm
COPD	III	Ex	5 to 10	40 to 60	m	60	Squamous cell carcinoma	≈8.5 mm
COPD	II	Ex	> 20	21 to 40	m	67	Squamous cell carcinoma	≈11 mm
COPD	II	Ex	6 to 10	> 60	m	70	Carcinoma	≈15 mm
Control	NA	Ex	> 20	20 to 40	m	72	Primary histiocytic sarcoma	≈15 mm
Control	NA	Ex	5 to 10	40 to 60	m	71	Pleomorphic carcinoma	≈8 mm
Control	NA	Ex	11 to 20	11 to 20	m	69	Adenocarcinoma	≈5 mm

### 2.2. Culture of primary human bronchial epithelial cells

The primary human bronchial epithelial cells were expanded and cultured as described previously (Berthold et al., 2022; Mastalerz et al., 2022). For more details, refer to the online Supplementary material.

### 2.3. Lectin-based and immunohistochemical fluorescent staining

#### 2.3.1. Sample preparation

Formalin-fixed, paraffin-embedded lung sections were deparaffinized with xylene, rehydrated in 100–50% ethanol, and rinsed in tap water. Antigen retrieval was performed in 10 mM citrate buffer, pH 6, with heat (125°C for 30 s, followed by 90°C for 10 s). Slides were washed 3 times in 1 × Tris-buffered saline. Paraformaldehyde-fixed phBECs on membranes were washed once with PBS and cut into 1/6 of the whole membrane with a scalpel.

#### 2.3.2. Lectin-based histochemical staining for detection of human-type flu receptor

To prepare a negative control, slides/phBECs on membranes were incubated with neuraminidase from *Arthrobacter ureafaciens* (Merck KGaA, #10269611001) with a 1:100 dilution in acetic acid buffer, pH 5.5, for 20 h at 37°C. Slides/phBECs on membranes incubated with and without neuraminidase were then blocked with 5% BSA in PBS for 30 min at room temperature and incubated overnight at 4°C with fluorescein isothiocyanate-conjugated *Sambucus nigra* lectin (FITC-SNA) (SNAI, US Bio, S0071-20A.2) (1:50 in 5% BSA in PBS, final concentration 40 μg/ml).

#### 2.3.3. Immunohistochemistry

Slides/phBECs on membranes were incubated overnight at 4°C with antibodies against the avian-type flu receptor, acetylated tubulin (acTub), mucin 5AC (MUC5AC), club cell-specific protein 10 (CC10), or cytokeratin 5 (CK5) diluted in 5% BSA in PBS. After washing three times with PBS, slides/phBECs on membranes were incubated with secondary antibodies and 4′,6-diamidino-2-phenylidole (DAPI) (1:2,000 in 5% BSA in PBS) for 1 h at room temperature. For phBECs on membranes, cells were permeabilized in 0.2% Triton X-100 in PBS for 10 min at room temperature prior to applying primary antibodies for differentiation markers. Permeabilization was not performed to detect flu receptors, since the cell-surface display is important for the virus to attach. Primary and secondary antibodies are listed in Supplementary Tables 2, 3, respectively. After washing 3 times with PBS, lung sections on glass slides were covered with mounting medium (DAKO). Membranes with phBECs were put on glass slides, covered in mounting medium, and coverslips were placed on top. Fluorescent microscopy was performed using the Axiovert II (Carl Zeiss AG, Oberkochen, Germany). For phBECs, immunofluorescence quantification was performed using the Imaris 7.4.0 software (Bitplane). Briefly, z-stack images of stained transwell membranes were generated by fluorescent microscopy, and individual cells were analyzed for the positivity of specific markers, largely as described previously (Schamberger et al., 2015; Mastalerz et al., 2022). For patient lung sections, quantification was performed using NIS Elements version 5.41.00 (Nikon Solutions Co., Ltd.) as follows: the apical area of the bronchus was selected above the bronchial epithelial nuclear layer and along the apical surface to yield the bronchial region of interest (ROI). Pneumocytes were selected in three randomly selected alveolar walls to yield the alveolar/pneumocyte ROI. Areas positive for the human-type flu receptor (SNA, green) or avian-type flu receptor (HYB4, red) were detected by the same threshold in all samples and recorded as binary areas. Data are given as binary area/ROI area × 100%.

### 2.4. Transepithelial electrical resistance measurements

The transepithelial electrical resistance (TEER) measurements were largely performed as described previously (Mastalerz et al., 2022). For more details, refer to the online Supplementary material.

### 2.5. Infection of fully differentiated phBECs

Transwell plates were placed in the 37°C incubator for 30 min with 500 μl of pre-warmed HBSS on the apical side. Mucus was carefully removed by pipetting up and down in each insert 5 times, followed by aspiration of HBSS and the addition of another 500 μl of HBSS on the apical side. This cycle was repeated for a total of 5 times to completely remove mucus. To each well, 1.25 × 10^5^ TCID_50_ PR8 viruses in 100 μl of HBSS, or 100 μl of HBSS for mock infection, was added to the apical side of inserts (Zhang et al., 2005; Newby et al., 2006). The infectious dose was adapted from the procedure described by Wu et al. (2022). If all the cells in the insert were exposed to and susceptible to influenza A virus infection, this would correspond to a multiplicity of infection (MOI) of approximately 0.1. However, this MOI has to be seen in the context of three-dimensional cultures composed of different cell types (Newby et al., 2006): compared with two-dimensional cultures in which MOI indicates the number of infectious viral particles that can infect each cell, the organotypic bronchoepithelium includes multiple cell types, with basal cells not facing the apical surface. In addition, not all cell types express influenza A virus receptors. Following infection, plates were gently rocked several times and incubated at 37°C for 2 h. The apical side was gently washed with 500 μl of pre-warmed HBSS two times to remove unbound viral particles, and another 500 μl of HBSS was added and collected for viral titration for day 0. The basolateral side was washed two times with 1 ml of pre-warmed HBSS, and 1 ml of pre-warmed PneumaCult-ALI medium was added. For sample collection on days 1 and 3 post-infection, 500 μl of HBSS was added to the apical side of inserts, and plates were incubated for 30 min at 37°C in a humidified cell incubator at 37°C with 95% air and 5% CO_2_. After gently pipetting up and down 5 times, apical washes and phBECs on membranes were collected and stored at −80°C until RNA and protein extraction.

### 2.6. Quantitative mass spectrometry in data-independent acquisition mode

Equal total protein amounts (10 μg) of the cell lysate were digested with a modified filter-aided sample preparation procedure (Wisniewski et al., 2009) using Lys-C and trypsin as proteases and Microcon^®^ centrifugal filters (Sartorius Vivacon 500, 30 kDa) for buffer exchange and on-filter digest. Approximately 0.5 μg of peptides per sample were measured in a randomized manner on a Q-Exactive™ HF mass spectrometer online coupled to an Ultimate 3000 nano-RSLC (Thermo Scientific) in data-independent acquisition (DIA) mode as described previously (Lepper et al., 2018; Mattugini et al., 2018). Briefly, peptides were automatically loaded on a trap column [300 μm inner diameter (ID) × 5 mm, Acclaim PepMap100 C18, 5 μm, 100 Å, LC Packings] prior to C18 reversed phase chromatography on the analytical column (nanoEase MZ HSS T3 Column, 100 Å, 1.8 μm, 75 μm × 250 mm, Waters) at 250 nl/min flow rate in a 105 min non-linear acetonitrile gradient from 3 to 40% in 0.1% formic acid. Profile precursor spectra from 300 to 1,650 m/z were recorded at 120,000 resolution with an automatic gain control (AGC) target of 3e6 and a maximum injection time of 120 ms. Subsequently, fragment spectra were recorded in 37 overlapping DIA isolation windows of variable size covering a total of 300–1,650 m/z, each at 30,000 resolution with an AGC target of 3e6 and a normalized collision energy of 28. The recorded raw files were analyzed using the Spectronaut Pulsar software (Biognosys) (Bruderer et al., 2015) with a peptide and protein identification false discovery rate setting of < 1%, using an in-house human spectral library that was generated using Proteome Discoverer 2.1 (Thermo Scientific), the Byonic search engine (Protein Metrics), and the Swissprot Human database (release 2017_02). Quantification was based on MS2 area levels of all unique peptides per protein fulfilling the percentile 0.25 setting. Normalized protein quantifications were exported and used for calculations of fold changes and significance values. The mass spectrometry proteomics data have been deposited to the ProteomeXchange Consortium *via* the PRIDE (Perez-Riverol et al., 2022) partner repository with the data set identifier PXD031461.

### 2.7. Statistical analysis of quantitative mass spectrometry data

For the whole protein expression analysis, we used R (version 4.0.4). The data was pre-processed by variance stabilization and normalization (vsn package) (Huber et al., 2002). A reduction in the dimension of the data using tSNE (Krijthe, 2015) allowed us to identify the different patients as a batch effect. The data were corrected from the patient effect using ComBat (Leek et al., 2012, 2020), and the interaction between time and condition as covariates. Due to the induced correlation structure in the corrected data, ComBat might lead to the exaggerated significance and, therefore, to higher FDR (Li et al., 2021). This might artificially increase the effect of condition and time when correcting the data from the batch effect. For protein expression analysis, we used the R package limma (Ritchie et al., 2015). We specified a generalized linear mixed model with a random intercept for patients and the interaction between condition and time as a fixed effect. For sensitivity analysis, we additionally built the model with adjustment for health state, which revealed very similar results.

### 2.8. Gene expression analysis by qRT-PCR and Western blot

Real-time quantitative reverse transcription polymerase chain reaction (qRT-PCR) and Western blot analysis were performed as described previously (Mastalerz et al., 2022). For more details including antibodies used for Western Blot analysis (Supplementary Tables 2, 3) and primers used for qRT-PCR (Supplementary Table 4), please refer to the online Supplementary material.

## 3. Results

### 3.1. The human-type flu receptor is detected more frequently than the avian-type in human lung sections and organotypic bronchoepithelia

**FIGURE 1:**
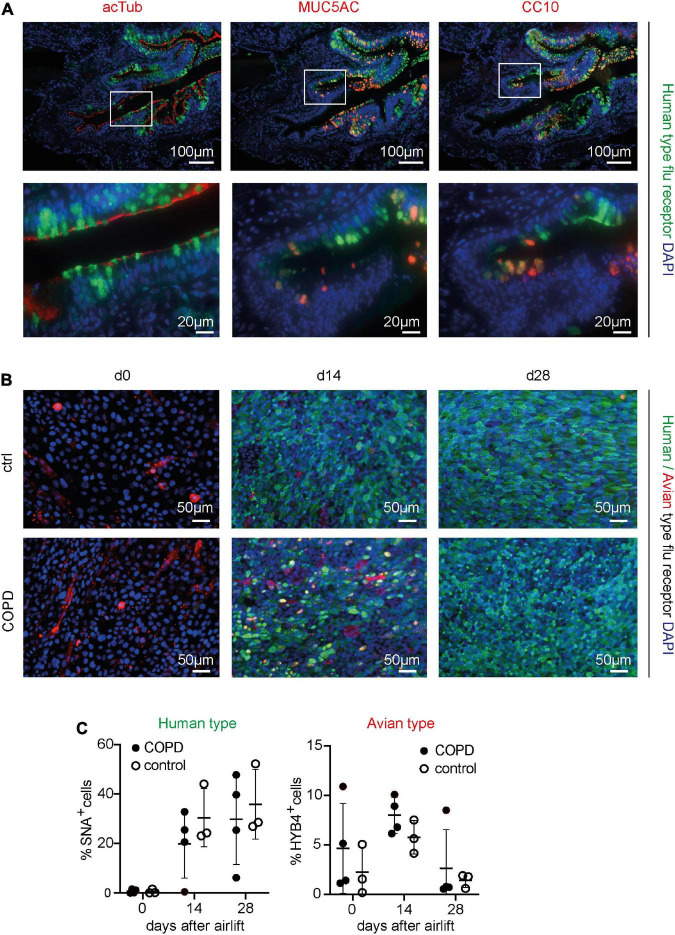
Detection of the human- and avian-type flu receptors in lung sections and organotypic bronchoepithelium from patients with chronic obstructive pulmonary disease (COPD) and controls. Human lung sections (COPD *n* = 5, control *n* = 5) and primary human bronchial epithelial cells (phBECs) from patients with COPD (*n* = 4) and controls (*n* = 3) were stained with Sambucus Nigra lectin (SNA) that specifically binds to sialic acids bound to galactose by the α2,6 linkage, SAα2,6Gal, the human-type flu receptor, and a monoclonal antibody (clone HYB4) that specifically binds to SAα2,3Gal, the avian-type flu receptor. **(A)** Detection of human-type flu receptor (green) in co-staining with antibodies directed toward acetylated tubulin (acTub), mucin 5AC (MUC5AC), or club cell-specific protein CC10 (red), for the identification of ciliated, goblet, or club cells, respectively, in formalin-fixed, paraffin-embedded human lung sections. The example shown is from a COPD lung explant. **(B)** Detection of human-type flu receptor (green) and avian-type flu receptor (red) in phBECs from COPD and control on days 0, 14, and 28 after airlift. **(C)** Quantification of the human- and avian-type flu receptors in phBECs on days 0, 14, and 28 after airlift. Panel **(A)** shows representative images for *n* = 5 (COPD) and *n* = 5 (control lung sections). Panel **(B)** shows representative images for *n* = 3 (control phBECs; here second control, see Table 1) and *n* = 4 (COPD phBECs; here GOLD stage III-derived phBECs); quantification of those data is given in panel **(C)**. For panel **(C)**, statistical analysis was performed using an unpaired, two-sided Mann–Whitney *U* test, but with a cutoff value of *p* < 0.05, no statistically significant differences were observed.

First, to assess the display of human- and avian-type flu receptors in the peripheral airway epithelium of patients with COPD and controls, formalin-fixed, paraffin-embedded lung sections (COPD *n* = 5, control *n* = 5) were stained with (1) FITC-SNA, which specifically binds to sialic acids bound to galactose by the α2,6 linkage (SAα2,6Gal), the human-type flu receptor (Ito et al., 1997) and (2) a monoclonal antibody (clone HYB4) that specifically binds to SAα2,3Gal, the avian-type flu receptor (Hidari et al., 2013). Pretreatment of a serial section with neuraminidase, an enzyme that cleaves sialic acids, resulted in a drastic reduction of the SNA signal, validating the specificity of the lectin-based histochemical staining (Supplementary Figure 1A). In the bronchial region, the human-type flu receptor was much more frequently detected than the avian-type flu receptor in patients with COPD and controls, while in alveolar regions, human- and avian-type flu receptors were displayed at similar levels (Supplementary Figures 1B, 2A, B). Despite considerable heterogeneity of flu receptor display across the samples, our results indicate that the levels of both humans- and avian-type flu receptors are decreased in patients with COPD compared with controls (Supplementary Figure 2). Staining with SNA targeting the human-type flu receptor in combination with antibodies against acetylated tubulin (acTub), mucin 5AC (MUC5AC), club cell-specific protein 10 (CC10), and cytokeratin 5 (CK5) demonstrated that the human-type flu receptor was not displayed by ciliated or basal cells, but rather by goblet and club cells (Figure 1A, Supplementary Figure 1B). The very infrequently detected avian-type flu receptor was not or little displayed by ciliated cells, never by the club or basal cells, but maybe by goblet cells (Supplementary Figure 1B). The latter was not directly assessed because both anti-MUC5AC and anti-SAα2,3Gal antibodies were of mouse origin (Supplementary Figure 1B).

Having confirmed the expression of flu receptors in small airways of patients with COPD and controls in lung sections, we made use of the organotypic model of phBECs differentiated at ALI. PhBECs from patients with COPD (*n* = 4) and controls (*n* = 3), matched for age and sex (Table 1), were differentiated for 28 days at ALI. Males were chosen considering the gender-specific prevalence of COPD (Ntritsos et al., 2018) as well as in efforts to reduce sample heterogeneity. Again, the specificity of lectin histochemistry in phBECs was confirmed by pretreating cells with neuraminidase (Supplementary Figure 1C). On ALI day 0, where the culture consists exclusively of basal cells, almost no expression of the human-type receptor was observed. The avian-type flu receptor, in contrast, was observed in all controls and patients with COPD at this time point, even if in relatively few cells only (up to 11%) (Figures 1B, C). On ALI days 14 and 28, in parallel to the emergence of all major differentiated cell types, the number of human-type flu receptor-positive cells increased in phBECs from controls and patients with COPD (Figures 1B, C). The avian-type receptor was still detectable in all cultures at similar levels at an intermediate time point of differentiation (day 14) but mostly lost at day 28 (Figure 1C).

As opposed to the tissue section staining, flu receptor display was similar for COPD- and control-derived cells *in vitro*. The much higher prevalence of human-type to avian-type flu receptors, however, agreed well with our observations in human lung sections, supporting the usefulness of our *in vitro* model for studies with influenza A virus infection where flu receptors serve as a major determinant of host susceptibility.

### 3.2. Patients with COPD- and control-derived cells show similar differentiation potential

The expression of differentiation markers assessed by immunofluorescence and qRT-PCR showed similar differentiation potential in cells from patients with COPD and controls. TEER value increased and reached a similar value on day 28 (Supplementary Figure 3). Ciliated cells continued to increase until day 28, goblet cells increased on day 14 and decreased on day 28, and club cells continued to increase until day 28 (Supplementary Figures 4A, B). *FOXJ1*, encoding a transcription factor specifically required for motile cilia, *MUC5AC*, and *SCGB1A1* (*CC10*) were expressed at similar levels on days 14 and 28 (unpaired, two-sided Mann–Whitney *U* test, *p* > 0.05) (Supplementary Figure 4C). PhBECs from patients with COPD tends to show a more heterogeneous expression of differentiation markers compared with those from controls.

### 3.3. COPD- and control-derived fully differentiated phBECs display largely similar protein profiles pre-infection

**FIGURE 2:**
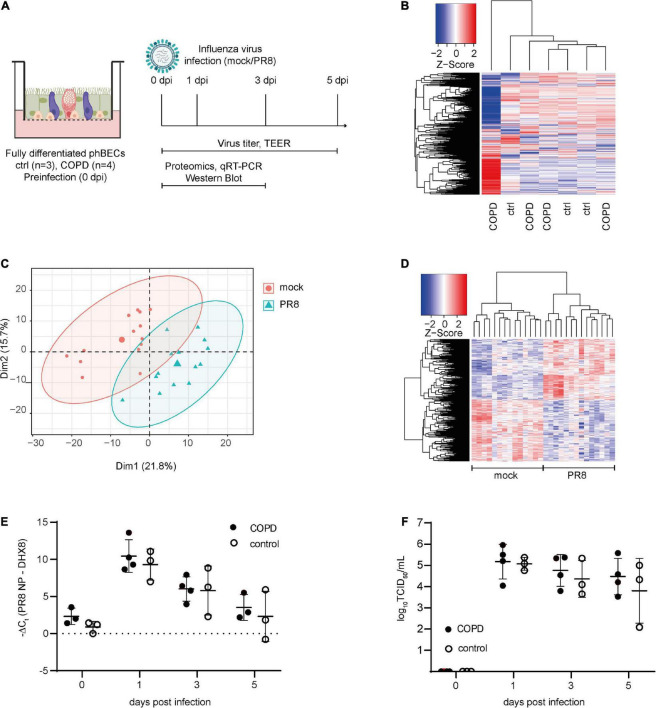
Proteomics pre- and post-influenza virus infection in fully differentiated primary human bronchial epithelial cells (phBECs). PhBECs from patients with chronic obstructive pulmonary disease (COPD) (*n* = 4) and controls (*n* = 3) were differentiated for 28 days at the air–liquid interface and either mock-infected or infected with influenza A virus PR8. Cells were harvested for proteomics on day 28 after airlift (pre-infection), and on days 1 and 3 post-infection. To examine viral replication, cells and apical washes were harvested on day 0 (after incubating the cells with the virus to allow attachment, and after removing the unattached virus by washing) and on days 1, 3, and 5 post-infection. **(A)** Scheme of culture, infection, and sample analysis. An illustration was created with biorender.com. TEER, transepithelial electrical resistance. **(B)** Heatmap of proteomics pre-infection. The protein profile of the COPD GOLD stage IV-derived phBECs is shown in the outermost left lane. **(C)** Principal component analysis of proteomics post-infection. **(D)** Heatmap of significantly altered proteins post-infection with all patients/controls and all time points (PR8 vs. mock, *n* = 7). A protein was considered to be differentially expressed if the comparison resulted in a false discovery rate (FDR) of less than 5% with the Benjamini–Hochberg (BH) correction to correct multiple testing. **(E)** Total RNA was extracted from cells and mRNA levels of PR8 nucleoprotein transcripts were examined by RT-qPCR. DHX8 was used as a housekeeping gene. **(F)** Infectious viral particles in apical washes were quantified by an end point dilution assay. TCID_50_: 50% tissue culture infectious dose. For panels **(E,F)**, statistical analysis was performed using an unpaired, two-sided Mann–Whitney *U* test, but with a cutoff point of *p* < 0.05, no statistically significant differences were observed.

To compare the protein expression in fully differentiated phBECs from patients with COPD and controls, cells were collected on ALI day 28 (Figure 2A) and subjected to proteomic analysis. The heatmap displaying overall protein changes in all subjects demonstrated that COPD- and control-derived phBECs did not cluster separately. Interestingly, however, the COPD GOLD stage IV-derived phBECs without a tumor background stood out with a unique protein profile (Figure 2B).

### 3.4. PhBECs derived from a COPD GOLD stage IV patient show distinct COPD-like features

Having observed that the COPD GOLD stage IV-derived phBECs displayed a unique protein profile compared to control and COPD GOLD stage II/III phBECs, we assessed to which extent this may reflect a more advanced COPD airway phenotype. Indeed, in particular, at day 28, the phBECs derived from the GOLD stage IV patient (*n* = 1) displayed a striking COPD-like phenotype in comparison to the control and less severe stage-derived cells: cells from that patient showed a lower TEER value, reduced levels of human-type flu receptor, and fewer ciliated and more goblet cells (Supplementary Figure 5A–C). Finally, from a panel of 20 genes known to be upregulated in COPD airway epithelial cells (Steiling et al., 2013; Wei et al., 2015), 14 showed increased levels in the GOLD stage IV phBECs as compared to the GOLD stage II/III and control phBECs (Supplementary Figure 5D). However, following infection, the PR8 virus still replicated similarly in these cells (Supplementary Figure 5E), indicating little biological influence of these features on infection.

### 3.5. Viral replication after influenza A virus infection does not differ in COPD- and control-derived fully differentiated phBECs

To evaluate the susceptibility and response of fully differentiated phBECs derived from patients with COPD and controls to influenza A virus infection, cells were infected with PR8, a widely used strain in *in vitro* studies (Kroeker et al., 2012, 2013; Short et al., 2016; Han et al., 2018). On ALI day 28, phBECs were infected with PR8 (Figure 2A) and proteomes were analyzed at days 1 and 3 post-infection (Figures 2C, D). Viral RNA of nucleoprotein within the cells and infectious viral particles in apical washes peaked at day 1 in phBECs from patients with COPD and controls and from then gradually decreased, with no statistical differences at any time point (unpaired, two-sided Mann–Whitney *U* test, Figures 2E, F).

### 3.6. COPD- and control-derived fully differentiated phBECs display similar protein profiles post-influenza A virus infection

Cellular protein was collected from phBECs on days 1 and 3 post-infection and subjected to proteomic analysis (Figure 2A). Principal component analysis (PCA) and a heatmap displaying the significantly altered proteins showed a clear separation between PR8- and mock-infected cells (Figures 2C, D), but not between disease origin (health state: COPD vs. control). An alternative dimensionality reduction method, t-Distributed Stochastic Neighbor Embedding (t-SNE), also indicated that only the condition effect (PR8-infected vs. mock-infected) separated the two groups, but not disease origin (COPD vs. control), time (day 1 vs. day 3 post-infection), or patients’ origin (Supplementary Figure 6). Moreover, the heatmap of significantly altered proteins on days 1 and 3 post-infection also did not indicate any separation between disease origin, and we found no significant changes in influenza virus-induced gene expression between control- and COPD-derived cells (Supplementary Figures 7, 8).

### 3.7. PhBECs display a robust antiviral response post-influenza A virus infection and upregulate several novel influenza virus-induced proteins

**FIGURE 3:**
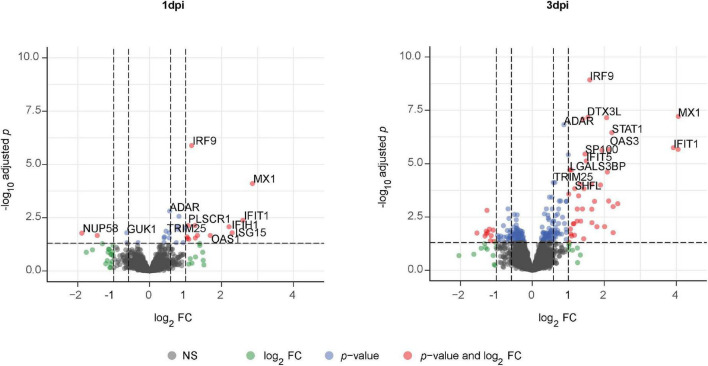
Volcano plot of significantly altered proteins in fully differentiated primary human bronchial epithelial cells (phBECs) on days 1 and 3 post-influenza virus infection. Fully differentiated phBECs from patients with chronic obstructive pulmonary disease (COPD) (*n* = 4) and controls (*n* = 3) were either mock-infected or infected with influenza A virus PR8. Cells were harvested for proteomics on days 1 and 3 post-infection (dpi). Volcano plot of significantly altered proteins (PR8/mock) on days 1 **(left)** and 3 **(right)** post-infection, independent of the disease state. A protein was considered to be differentially expressed if the comparison resulted in a false discovery rate (FDR) of less than 5% with the Benjamini–Hochberg (BH) correction to correct multiple testing.

**FIGURE 4:**
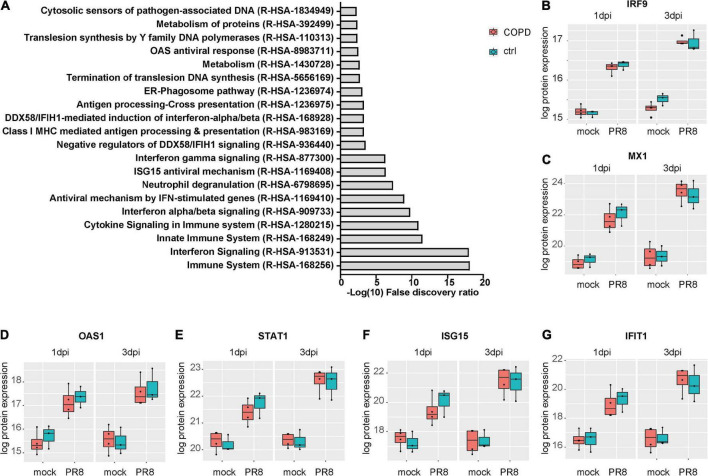
Upregulation of interferon-regulated pathways and proteins in fully differentiated primary human bronchial epithelial cells (phBECs) independent of disease state post-influenza virus infection. **(A)** Pathway analysis performed using the tool PANTHER and all 290 differentially expressed proteins post-infection, independent of disease state and taking account of both time points. **(B–G)** Box plots of interferon-regulated proteins depicting the median (middle horizontal line), the 25 and 75% percentile (lower and upper edge of the box), and the range between largest and smallest value up to a maximum of 1.5 × interquartile range (vertical lines). Data points beyond that range are regarded as outliers and are shown as individual points. IRF9, interferon-regulatory factor 9; MX1, Interferon-induced GTP-binding protein Mx1; OAS1, 2’–5’-oligoadenylate synthase 1; STAT1, signal transducer and activator of transcription 1-α/β; ISG15, ubiquitin-like protein ISG15; IFIT1, interferon-induced protein with tetratricopeptide repeats 1.

**FIGURE 5:**
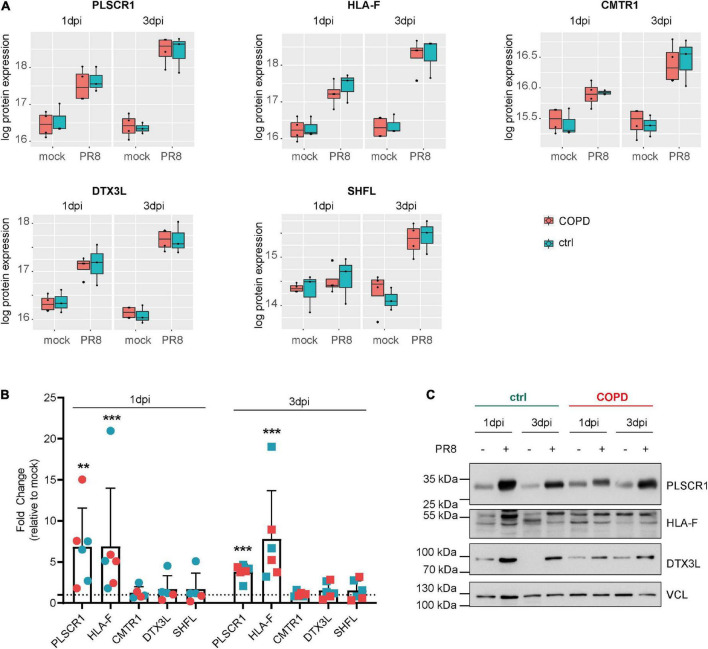
Novel proteins upregulated in fully differentiated primary human bronchial epithelial cells (phBECs) independent of disease state post-influenza virus infection. **(A)** Box plots of significantly upregulated novel proteins in fully differentiated phBECs on days 1 and 3 post-influenza virus infection (dpi). PLSCR1, phospholipid scramblase 1; HLA-F, human leukocyte antigen class I histocompatibility antigen, α chain F; CMTR1, cap-specific mRNA (nucleoside-2′-*O*-)-methyltransferase 1; DTX3L, E3 ubiquitin–protein ligase DTX3L; SHFL, shiftless antiviral inhibitor of ribosomal frameshifting protein. Box plots depict the median (middle horizontal line), the 25 and 75% percentile (lower and upper edge of the box), and the range between the largest and smallest value up to a maximum of 1.5 × interquartile range (vertical lines). Data points beyond that range are regarded as outliers and are shown as individual points. **(B)** mRNA expression of novel genes 1 and 3 dpi (PR8/mock). For some COPD phBECs samples, the obtained amount of RNA was insufficient to cover all qPCR reactions, restricting the COPD-derived samples to *n* = 3 and *n* = 2 on 1 dpi. Symbol legend (in analogy to panels A and C): Cyan squares, ctrl samples; red squares, COPD samples. **(C)** Western blots of PLSCR1, HLA-F, and DTX3L. VCL (vinculin) was used as a loading control. Irrelevant parts of the Western blot images were cropped to only present the areas of interest. Uncropped Western blot images are given in Supplementary Figure 9.

Given the absence of disease-specific effects, we focused on differences between PR8- and mock-infected cells, independent of the disease origin (*n* = 7). In this analysis, 31 and 234 proteins were differentially expressed (adj. *p* < 0.05) on day 1 and day 3 post-infection (PR8/mock), respectively, with a robust upregulation of interferon (IFN)-regulated genes (Figure 3, Supplementary Figures 7, 8, and Supplementary Data file “Protein List,” data sheets “ConditionsDay1” and “ConditionsDay3”). Taking into account both time points, 290 proteins were differentially expressed post-infection (adj. *p* < 0.05) among the total 4,341 variables (PR8/mock; Supplementary data file “Protein List,” data sheet “ConditionsOverallTime”). Pathway enrichment analysis of those 290 proteins demonstrated that variants of the (innate) immune system and cytokine signaling, in particular IFN signaling, were the dominating top enriched pathways (Figure 4A). For instance, a strong upregulation of established IFN-regulated genes like interferon-regulatory factor 9 (IRF9), interferon-induced GTP-binding protein Mx1 (MX1), 2′–5′-oligoadenylate synthase 1 (OAS1), signal transducer and activator of transcription 1-α/β (STAT1), ubiquitin-like protein ISG15 (ISG15), and interferon-induced protein with tetratricopeptide repeats 1 (IFIT1) was observed (Figures 4B–G). In addition to these drastically upregulated proteins demonstrating a robust antiviral response, several novel proteins appeared as significantly upregulated (adj. *p* < 0.05). Those proteins were phospholipid scramblase 1 (PLSCR1), human leukocyte antigen class I histocompatibility antigen, α chain F (HLA-F), cap-specific mRNA (nucleoside-2′-*O*-)-methyltransferase 1 (CMTR1), E3 ubiquitin–protein ligase DTX3L (DTX3L), and shiftless antiviral inhibitor of ribosomal frameshifting protein SHFL, previously called RyDEN (c19orf66) (Figure 5A). Upregulation of all these genes was assessed by RT-qPCR (Figure 5B) and, for PLSCR1, HLA-F, and DTX3L, by Western blot analysis (Figure 5C, Supplementary Figure 9). Upregulation on protein level was validated for all three proteins assessed, while upregulation on transcript level was only observed for PLSCR1 and HLA-F, demonstrating significant post-transcriptional regulation mechanisms.

## 4. Discussion

Studies comparing proteomes between COPD- and control-derived phBECs in fully differentiated organotypic bronchoepithelia pre- and post-influenza virus infection are lacking. Performing such a comparison, we have not found disease-dependent changes. Independent of disease status, we have, however, observed a robust IFN-mediated antiviral response and identified several upregulated proteins following PR8 infection, which have not or only recently been linked to the host response to the influenza virus. Notably, those proteins have not been reported in studies where undifferentiated primary normal human bronchotracheal epithelial cells were infected with influenza virus PR8 (Kroeker et al., 2012, 2013). Our study has thus identified novel influenza-regulated proteins in the fully differentiated bronchial epithelium that may qualify as targets to modulate the pathogenicity of infection and acute exacerbation caused by the influenza virus.

In agreement with previously published reports, we observed that the human-type flu receptor is much more frequent in human airways and fully differentiated phBECs than the avian-type (Shinya et al., 2006; Davis et al., 2015; Hsu et al., 2016) and predominantly localized on bronchial secretory cells (Matrosovich et al., 2004). The specific bronchial epithelial cell types presenting the avian-type flu receptor, however, remain poorly identified. While the display of the avian-type flu receptor on ciliated cells has been reported by others (Matrosovich et al., 2004), our results do not provide evidence for this (Supplementary Figure 1B). However, it must be acknowledged that the overall low presentation of the avian-type flu receptor in the human airway (Matrosovich et al., 2004; Nicholls et al., 2007; Davis et al., 2015) represents a challenge for a systematic assessment of the cell types displaying it. In addition, discrepancies between studies may also stem from different detection systems and protocols. For instance, it has been reported that *Maackia amurensis* agglutinin (MAA) lectins, frequently used to detect the avian-type flu receptor, differ in their binding affinity to SAα2,3 depending on supplier and MAA isoform used (Matrosovich et al., 2004; Nicholls et al., 2007). Here, we took advantage of a more recently developed specific monoclonal mouse antibody (HYB4) (Hidari et al., 2013; Supplementary Figure 1B), which allowed for circumventing the known variability in lectin binding but restricted our choice of specific antibodies to co-stain with. Therefore, even if our results, by way of exclusion, suggest display of the avian-type receptor by goblet cells in human bronchial epithelium, we have not been able to directly demonstrate this. The cell types displaying the avian-type flu receptor thus remain elusive and need further investigation.

Interestingly, despite considerable patient heterogeneity, fluorescent staining-based quantification suggested that flu receptors overall may be downregulated in patients with COPD. Also, the thickened mucus layer and increased expression of mucins in patients with COPD may protect them from influenza virus infection both because cell membranes are less accessible and because heavily sialylated mucins act as decoy receptors (Ehre et al., 2012; Wallace et al., 2021). Here, before *in vitro* infection of phBECs, we removed the mucus layer in effort to focus on cell-associated differences in virus susceptibility. Furthermore, as opposed to flu receptors in patients’ epithelium, we did not see any significant changes in flu receptor display in COPD-derived vs. control-derived phBECs *in vitro*. These two aspects may explain why viral replication was similar for control- and COPD-derived phBECs *in vitro*. Flu receptor display not being conserved in COPD-derived cells *in vitro* could, on the one hand, be due to optimized growth and differentiation conditions that certainly do not reflect the microenvironment in COPD airways. On the other hand, a decrease in flu receptor display may also only manifest in later stages of COPD: while we mainly used COPD GOLD stage II/III-derived cells for differentiation, lung specimens used for staining and quantification of flu receptors *in vivo* were derived from end-stage patients with COPD. In support of this, human-type flu receptor display is drastically reduced in phBECs derived from a COPD GOLD stage IV patient (*n* = 1, Supplementary Figure 5B). Overall, these observations rather support a lower susceptibility of patients with COPD to influenza virus infection. However, interestingly, during the differentiation of phBECs, we regularly observed more avian-type receptors at early time points, where basal cells are overrepresented and where the culture thus reflects properties of a regenerating bronchial epithelium (Schamberger et al., 2015; Mastalerz et al., 2022). This suggests that, under conditions of acute airway injury and ongoing regeneration or in smoking-induced airway basal cell hyperplasia (Crystal, 2014), bronchial epithelial cells may be more susceptible to infection by the avian influenza viruses. These observations warrant further studies in models of airway regeneration and hyperplasia.

Comparisons between COPD- and control-derived phBECs have been done by direct transcriptomic analysis of bronchial brushings, identifying several differentially expressed genes that characterized COPD-derived phBECs (Steiling et al., 2013). It has also been shown that phBECs derived from GOLD stage IV patients differentiate differently relative to control cells, resulting in altered cell type compositions (Gohy et al., 2019). Here, we selected phBECs from age-, sex-, and smoking history-matched control and COPD lungs (GOLD stage II/III), all isolated from non-tumorous airways in the context of a lung tumor resection, except for one GOLD stage IV specimen from an end-stage explant. However, in our differentiated organotypic bronchoepithelia, we did not find any statistically significant differences between COPD- and control-derived phBECs in terms of proteomes and differentiation potential. Identification of such differences may have been hampered by the following limitations in our study design: first, we included phBECs from different COPD GOLD stages, including mild-to-moderate COPD. Several large COPD cohort studies showed that the COPD GOLD stage is not necessarily a stable or progressive criterion (due to diagnosis by spirometry and work-related lung function diagnostics). Especially patients who initially meet the criteria for mild-to-moderate COPD are sometimes later diagnosed with lower COPD GOLD stages or even with normal lung function, or vice versa, over a series of annual visits (Aaron et al., 2017). This suggests reversibility of disease stage unless cells are from severe or very severe patients with COPD, which may in part be reflected by the capacity of airway epithelial cells to differentiate normally *in vitro* when given the appropriate growth factors. Second, given the known individual variations in patient-derived cells including a highly diverse medication history (Supplementary Table 1), the low number of control- and COPD-derived phBECs used in our study may have been insufficient to detect significant differences (Mindaye et al., 2017). Third, it cannot be completely excluded that tumor environment-derived factors may have ultimately masked COPD-specific effects for all but phBECs from the stage IV patient without a tumor background.

Although influenza A virus infection has been studied using proteomics in basal-like phBECs under submerged conditions (Kroeker et al., 2012, 2013), an advantage of our study is that we used fully differentiated phBECs, which reflect the cell type complexity of the bronchial epithelium *in vivo* and thus recapitulate the orchestration of numerous host factors better than undifferentiated cells or cell lines. Nevertheless, our *in vitro* model does not recapitulate all features of the bronchial epithelium *in vivo* including epithelial–immune cells or epithelial–mesenchymal crosstalk. Such interactions, however, may be key in the context of acute exacerbation and COPD progression (Barnes, 2019; Guo-Parke et al., 2020). Therefore, future studies on influenza virus infection should make use of phBECs derived from very severe or severe COPD GOLD stage lungs, which typically retain a disease phenotype *in vitro*, ideally in combination with non-tumorous and, in terms of age and sex, well-matched control samples. Furthermore, the influence of deregulated immune or mesenchymal cells should be studied in more advanced organoid co-culture models.

Next to a strong and mostly IFN signaling-related antiviral response, we discovered several influenza virus-induced proteins that have not or only recently been linked to influenza virus infection and may thus represent novel host factors in that context. PLSCR1, for instance, is an IFN-regulated gene with multiple functions including the regulation of viral uptake and replication by interacting with viral proteins at the plasma membrane as well as within the cytoplasm (Dal Col et al., 2022). The few studies that addressed its role in influenza virus infection have demonstrated binding to nucleoprotein NP and prevention of its nuclear translocation (Luo et al., 2018; Liu et al., 2022). Interestingly, PLSCR1 has also been described to be secreted and interact with extracellular matrix protein 1 (ECM1) in the dermal–epidermal junction of human skin (Merregaert et al., 2010). This raises the intriguing hypothesis that PLSCR1 could also play a role in epithelial–mesenchymal crosstalk and, for instance, promote small airway fibrosis upon influenza virus infection. Notably, small airway fibrosis is increasingly recognized as the major driver of COPD progression, but the underlying causative mechanisms remain poorly understood (Hogg et al., 2017; Barnes, 2019). Hence, further investigation is warranted to assess the role of this protein in the context of airway fibrosis.

Cap-specific mRNA (nucleoside-2’-O-)-methyltransferase 1 (CMTR1) and SHFL have been described as host factors that facilitate the expression of viral proteins. Upon influenza A virus infection, CMTR1 is required for efficient cap snatching and regulation of a cell’s autonomous immune response (Li et al., 2020). A function of SHFL has not yet been described in the context of influenza virus infection, but it is known to promote programmed-1 ribosomal frameshift (−1 PRF) in SARS-CoV-2 viral gene expression (Baggen et al., 2021; Sun et al., 2021). Importantly, an inhibitor of −1 PRF, merafloxacin, restricted SARS-CoV-2 replication in VERO E6 cells (Sun et al., 2021). Taken together, these observations warrant further studies into CMTR1 and SHFL as potential influenza virus host factors and targets to modulate influenza pathogenicity.

E3 ubiquitin-protein ligase DTX3L (DTX3L), an E3 ubiquitin–protein ligase, has been little investigated in the context of virus infection, except for a study that reports protein mono-ADP-ribosyltransferase PARP9 (PARP9)-DTX3L ubiquitin ligase to target host histone and viral protease to enhance IFN signaling (Zhang et al., 2015). It is known that the ubiquitin–proteasome system is important for the replication of influenza A viruses and that ubiquitination also plays an essential role in innate immunity to the influenza A virus (Rudnicka and Yamauchi, 2016). DTX3L may thus be a hitherto unidentified factor in this context.

Finally, recent studies have been revealing the significance of natural killer (NK) cells in maintaining immune system homeostasis and their involvement in COPD pathogenesis (Rao et al., 2021). NK cells, as innate immune cells, provide the first line of defense at the border of airway epithelium and the microenvironment. HLA-F, upregulated in our model upon influenza virus infection, has been the least investigated among the HLA class I family; it has only recently been demonstrated to exert an important role in immune regulation by presenting peptides and regulating immunity through interactions with NK cells (Dulberger et al., 2017; Lin and Yan, 2019). Interestingly, increased protein levels of HLA-F and interaction with NK cells have been indicated after infection with BK polyomavirus, HIV, and HCV (Lunemann et al., 2018; Kiani et al., 2019; Koyro et al., 2021). Our study indicates a role for HLA-F after influenza virus infection in the human airway, presumably by interaction with NK cells. Since the functions of NK cells in COPD have remained unclear (Rao et al., 2021), the differentiated organotypic bronchoepithelia might serve as a simplified model to unravel the interaction of NK cells with HLA-F upon viral infection.

## Conclusion

Our study demonstrated the dominant presentation of human-type vs. avian-type flu receptors in human lung sections and in fully differentiated phBECs. COPD- and control-derived cells showed similar differentiation potential, similar susceptibility to influenza A virus infection, and similar proteomes pre- and post-infection. Pooling all post-infection proteome data enabled us to identify novel influenza A virus-regulated proteins. Our results warrant further studies of these proteins’ functions in the context of the pathogenicity of infection and acute exacerbation caused by the influenza virus.

## Data availability statement

The datasets presented in this study can be found in online repositories. The names of the repository/repositories and accession number(s) can be found below: http://www.proteomexchange.org/, PXD031461.

## Ethics statement

The studies involving human participants were reviewed and approved by Ethics Committee of the Justus-Liebig-University School of Medicine, Gießen, Germany, Ethics Committee of the Ludwig-Maximilians University of Munich, Germany. The patients/participants provided their written informed consent to participate in this study.

## Author contributions

MN, HA, and CS-W conceived and designed the research and wrote the manuscript. HA and CS-W supervised the project. MN, CS-W, HA, AD, AC, and EH planned and performed the experiments and analyzed the data. JM-P and SH performed the proteomics and data analysis. HM and RL performed the statistical analysis on proteomics data. CR, AG, KK, JB, and AH provided the clinical samples, patient parameters, and resources. All authors read and approved the manuscript.
