# Supplementary material for: Quantitative proteomics of differentiated primary bronchial epithelial cells from chronic obstructive pulmonary disease and control identifies potential novel host factors post-influenza A virus infection
Source: Front Microbiol. 2023 Jan 11;13:957830. doi: 10.3389/fmicb.2022.957830 (PMC9875134; doi:10.3389/fmicb.2022.957830)
Supplement: Supplementary file 1 [file Data_Sheet_1.pdf]

Quantitative proteomics of differentiated primary bronchial epithelial cells from chronic obstructive pulmonary disease and control identifies potential novel host factors post-influenza virus infection

Misako Nakayama[1,2], Hannah Marchi[3,4], Anna Mikhailovna Dmitrieva[5], Ashesh Chakraborty[1], Juliane Merl-Pham[6], Elisabeth Hennen[1], Ronan le Gleut[3], Clemens Ruppert[7], Andreas Guenther[7], Kathrin Kahnert[8], Jürgen Behr[8], Anne Hilgendorff[1], Stefanie M. Hauck[6], Heiko Adler[5,9 *], Claudia Alma Staab-Weijnitz[1 *]

ONLINE DATA SUPPLEMENT

SUPPLEMENTARY MATERIAL AND METHODS

Influenza virus A/Puerto Rico/8/34 (PR8) stock production

Influenza virus A/Puerto Rico/8/34 (PR8) ($60x10^6$ PFU/mL) and MDCK II cells were a gift from Prof. Dr. med. Susanne Herold (University of Giessen and Marburg Lung Center). MDCK II cells (ATCC CRL-2936) are a subclone (derived by limited dilution cloning) of the parental cell line MDCK. They were cultured in T-75 flasks at 37°C 5% CO_2, in minimum essential media (MEM) (GIBCO, cat # 31095052) supplemented with 10% FCS (PAN Biotech, cat # P30-3702), penicillin and streptomycin (100 U/ml each) (GIBCO, cat # 15140-122). 20 μL of PR8 from Giessen was diluted up to 2 mL with MEM supplemented with 0.1% BSA, penicillin and streptomycin (100 U/ml each) and TPCK-trypsin (final concentration 5 μg/mL) (SIGMA, cat # T4549), and sterile-filtered through a 0.22 μm pore membrane. 500 μL of filtered PR8 was added onto almost confluent MDCK II cells, with 10 mL of the same media used to dilute the virus. Cells were incubated in a humidified cell incubator at 35°C with 95% air and 5% CO_2. After four days, a clear cytopathic effect (CPE) was seen with only 30-40% of cells remaining attached. Media was collected and centrifuged at 1300 rpm for 5 min, and supernatant was sterile-filtered to make PR8 master stocks. One master stock was titrated with MCDK II cells, which was 10^7 $TCID_{50}$/mL, and expanded once in MDCK II cells to make working stocks, which was 10^8 $TCID_{50}$/mL. Aliquots of working stock PR8 were stored at -80°C until use.

Virus quantification with an endpoint dilution assay

MDCK II cells were cultured on 96 well plates until confluent. They were washed once with 200 μL of MEM with 0.1 % BSA, penicillin and streptomycin (100 U/mL each). Master stock and working stock PR8 virus, and apical washes collected from phBECs cultured on Transwell inserts were used to make ten-fold dilutions in MEM with 0.1 % BSA, penicillin and streptomycin (100 U/mL each) supplemented with TPCK-trypsin (final concentration 5 μg/mL). Those ten-fold dilutions were added to wells in quadruplicate. CPE was examined under a phase contrast microscope 72 hours later. The dilution at which 50% of the wells showed CPE was used to calculate the median tissue

culture infectious dose ($TCID_{50}$) by the Reed and Muench method.

Culture of primary human bronchial epithelial cells (phBECs)

PhBECs, passage 1, were thawed, cultured on flasks coated with collagen-I (Merck KGaA; Darmstadt, Germany, C3867) in PneumaCult Ex-Plus medium (Stemcell Technologies; Köln, Germany, #05040) supplemented with hydrocortisone 21-hemisuccinate sodium salt (Merck KGaA; H2270) dissolved in 2% sodium chloride (final concentration of hydrocortisone 21-hemisuccinate sodium salt: 0.48 ug/ml), penicillin and streptomycin (100 U/ml each) (PCEP), in a humidified cell incubator at 37°C with 95% air and 5% CO_2. When cells reached nearly 80% confluency, cells were detached with detachment solution, and inhibition solution was added (Animal Component-Free Cell Dissociation Kit, Stemcell Technologies; #05426). Cells were suspended in PCEP for cell count. Trypan blue was added to identify dead cells. For differentiation of phBECs, 80,000-100,000 live cells at passage 2 were transferred to 12 mm Transwells with 0.4 µm pore polyester membrane inserts (12-well, Corning Inc; Corning, NY, #3460) coated with human placental collagen-IV (Merck KGaA; #C7521), filled with pre-warmed PCEP in the basal (1 mL) and apical (500 µL) side of the insert. When cells reached confluency, basal medium was substituted with PneumaCult-ALI medium (Stemcell Technologies; #05001) supplemented with heparin (Merck KGaA; H3149-10KU, final concentration: 4 µg/ml) and hydrocortisone 21-hemisuccinate sodium salt (0.48 µg/ml), and medium on the apical side of the insert was aspirated for culture at the air-liquid interface (ALI). ALI day 0 refers to the day when phBECs were airlifted. Basolateral media was changed every 2-3 days and mucus on the apical side was washed with HBSS (Thermo Fisher Scientific, Inc; Waltham, MA, #14065, diluted to 1X with distilled water) every week from ALI day 14. On ALI day 0, 14 and 28, two membranes were collected and stored at -80°C until protein and RNA extraction; one membrane was fixed in 4 % PFA for 1 hour at room temperature and stored in PBS at 4°C until immunohistochemistry/lectin staining.

Transepithelial electrical resistance (TEER) measurements

On ALI day 0, 7, 14, 21, 28 (pre-infection), TEER values were measured in every well by Millicell ERS-2 Voltohm meter and a STX01 chopstick electrode (Merck KGaA, Darmstadt, Germany). The number of wells measured at each timepoint was 28 to 36 wells pre-infection. For this, 500 µL of pre-warmed HBSS was added on the apical side and left for 10-15 min for the medium to reach room temperature. Three points per membrane was measured, and the average values were calculated, blank substracted, and multiplied by the membrane area (1.12 cm^2) to yield the final result in Ω x cm^2.

qRT-PCR

On ALI day 0, 14 and 28, one membrane was collected and stored at -80°C. Membranes were thawed on ice and RNA extraction was performed with RNeasy Plus Mini Kit (QIAGEN; Hilden, Germany, #74135) according to the manufacturer's protocol. RNA concentration was determined with NanoDrop TM 1000 spectrophotometer (NanoDrop Tech. Inc; Wilmington, DE) at 260 nm. Reverse transcription was performed with 1250 ng of RNA using High-Capacity cDNA Reverse Transcription Kit (Thermo Fisher Scientific, #4368813) according to the manufacturer's protocol. 12.5 ng of template cDNA in 2.5 µl of DNase/RNase-free water, 5 µL of LightCycler 480 DNA SYBR Green I Master (Roche; Basel, Switzerland, #04707516001), 1 µL of primer mix (2.5 pmol), and 1.5 µl of DNase/RNase-free water were mixed, and qRT-PCR was performed in a 96-well format using the Light Cycler LC480II instrument (Roche). Primers were obtained from Eurofins Genomics Germany GmbH (Ebersberg, Germany) and are listed in Supplementary Table 4.

Western blot analysis

PhBECs on membranes were thawed on ice and cells were scraped with 160 µL of lysis buffer (10 mM Tris-HCL pH 7.5, 1% sodium Deoxycholate and 1mM EDTA-Na) supplemented with 1X complete protease inhibitor cocktail and 1X phosphatase inhibitor cocktail (Merck KGaA, #11697498001 and #04906837001, respectively). Whole cell lysates were collected, incubated for 20-30 min on ice, centrifuged with 14,000 x g for 15 min at 4°C. Supernatant was collected and protein concentration was measured by BCA™ Protein Assay kit (Thermo Fisher Scientific, #23225) according to the manufacturer's instructions. 15-30 µg of protein was loaded and separated by a SDS-

PAGE gel and transferred to polyvinylidene difluoride membrane as previously described (Schamberger AC et al. 2015). Blots were blocked with 5% milk powder in Tris-buffered saline with 0.1% Tween, and incubated overnight at 4°C with primary antibodies against PLSCR1, DTX3L and HLA-F. They were then incubated with anti-rabbit IgG horseradish peroxidase. Blots were stripped with stripping buffer (Thermo Fisher Scientific, #46430) and incubated with vinculin. Immunoreactive proteins were visualized with chemiluminescent substrates (Thermo Scientific). Signals were detected with the ChemiDoc XRS^{+} Imaging system (Biorad; Hercules, CA). Quantification was performed with Image lab (version 5.2.1). Primary and secondary antibodies are listed in Supplementary Tables 2 and 3, respectively.

SUPPLEMENTARY FIGURES

Supplementary Figure 1: Lectin-based histochemical stainings for detection of the human-type and the avian-type flu receptor. (**A**) Validation of lectin-based histochemical staining to detect the human-type flu receptor in serial sections of formalin-fixed paraffin embedded lung sections. Lung sections (example shown here: COPD lung section) were pretreated in acetic acid buffer pH 5.5 with or without neuraminidase, an enzyme, which cleaves sialic acids. They were then incubated with fluorescein isothiocyanate (FITC)-conjugated *Sambucus Nigra* lectin (SNA) that binds specifically to SAα2,6Gal, the human-type flu receptor. Sections pretreated with neuraminidase and incubated with FITC-SNA resulted in a drastic reduction of the SNA signal, validating the specificity of the lectin-based histochemical staining. (**B**) Staining with SNA targeting the human-type flu receptor in combination with antibodies against acetylated tubulin (acTub), mucin 5AC (MUC5AC), club cell specific protein 10 (CC10), and cytokeratin 5 (CK5) in formalin-fixed paraffin embedded lung sections. Example shown is from a COPD patient. Upper row: the human-type flu receptor (green) was not displayed by ciliated (red) or basal (red) cells, but rather by goblet (red) and club cells (red). Lower row: the avian-type flu receptor (red) was very infrequently detected and was not or little displayed by ciliated cells (green), never by club or basal cells (green). Goblet cells was not directly assessed because both anti-MUC5AC and anti-SAα2,3Gal antibodies were of mouse origin. Yellow arrows point to flu receptors detected on the apical surface of bronchoepithelia since the apical surface is where the virus attaches to cells thus serving as a major determinant of host susceptibility. (**C**) Validation of lectin-based histochemical staining to detect the human-type flu receptor in fully differentiated phBECs cultured at the air-liquid interface. Fully differentiated phBECs (here derived from a control patient) on membranes were pretreated in acetic acid buffer pH 5.5 with or without neuraminidase, and incubated with FITC-SNA. PhBECs pretreated with neuraminidase and incubated with FITC-SNA resulted in a drastic reduction of the SNA signal, validating the specificity of the lectin-based histochemical staining in an organotypic bronchoepithelia. N/A, not assessed.

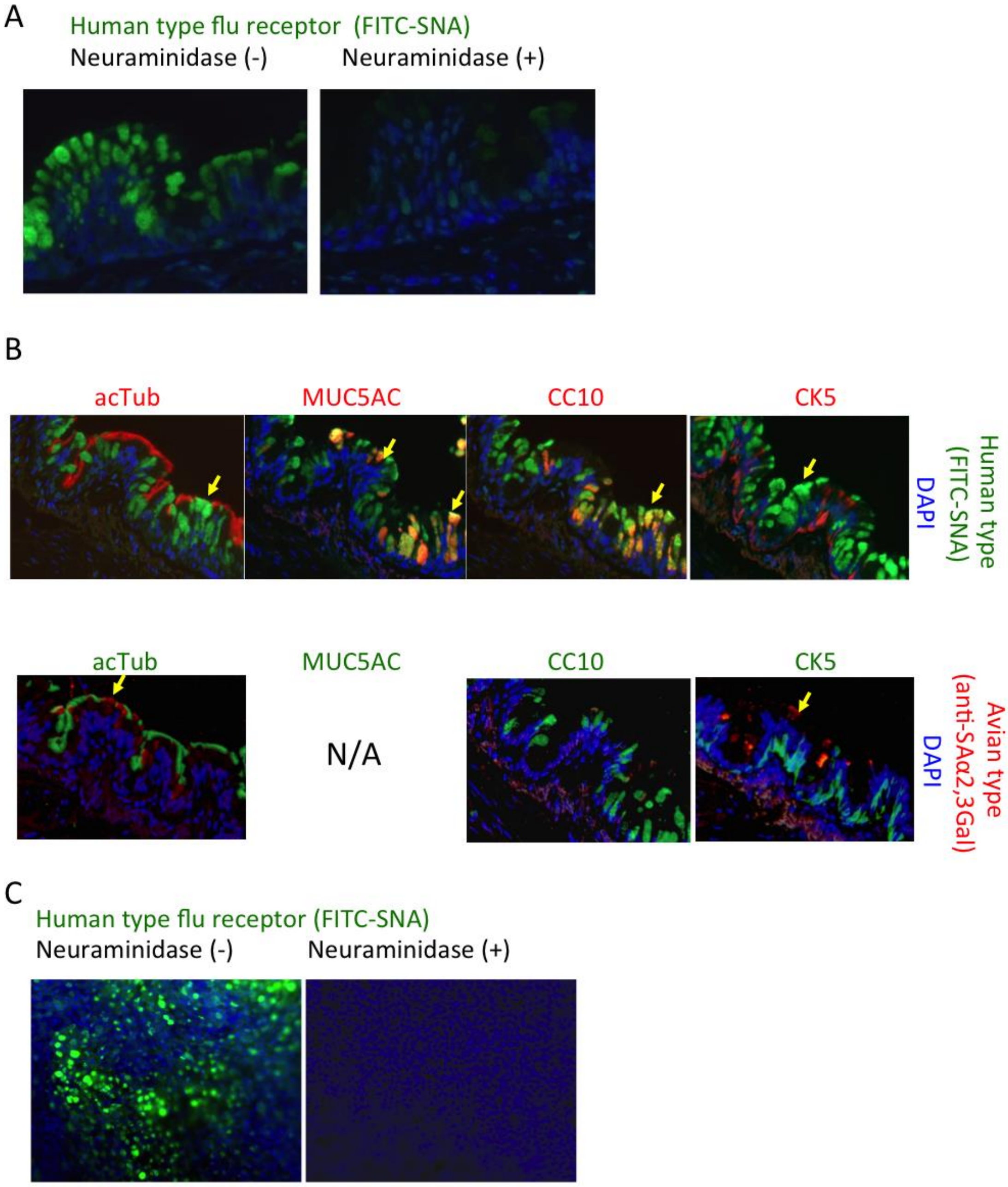
A
Human type flu receptor (FITC-SNA)
Neuraminidase (-)
Neuraminidase (+)
B
acTub
MUC5AC
CC10
CK5
Human type (FITC-SNA)
DAPI
acTub
MUC5AC
N/A
CC10
CK5
Avian type (anti-SAα2,3Gal)
DAPI
C
Human type flu receptor (FITC-SNA)
Neuraminidase (-)
Neuraminidase (+)

Supplementary Figure 2: Quantification of human- and avian-type influenza receptors in human lung sections. (**A**) Quantification was performed using NIS Elements version 5.41.00 (Nikon Solutions Co., Ltd) by selecting regions of interest (ROI) above the bronchial epithelial nuclear layer and along the apical surface for the bronchial compartment, and selecting three random alveolar walls for the alveolar compartment. Areas positive for the human-type flu receptor or avian-type flu receptor (identified in stainings as described in Figure 1 and Supplementary Figure 1) were detected by the same threshold in all samples and recorded as binary area. Example shown is from a COPD section. (**B**) Data is given as binary area/ROI area x 100 %. Statistical analysis was performed using a non-parametric Mann-Whitney test. *, $p<0.05$

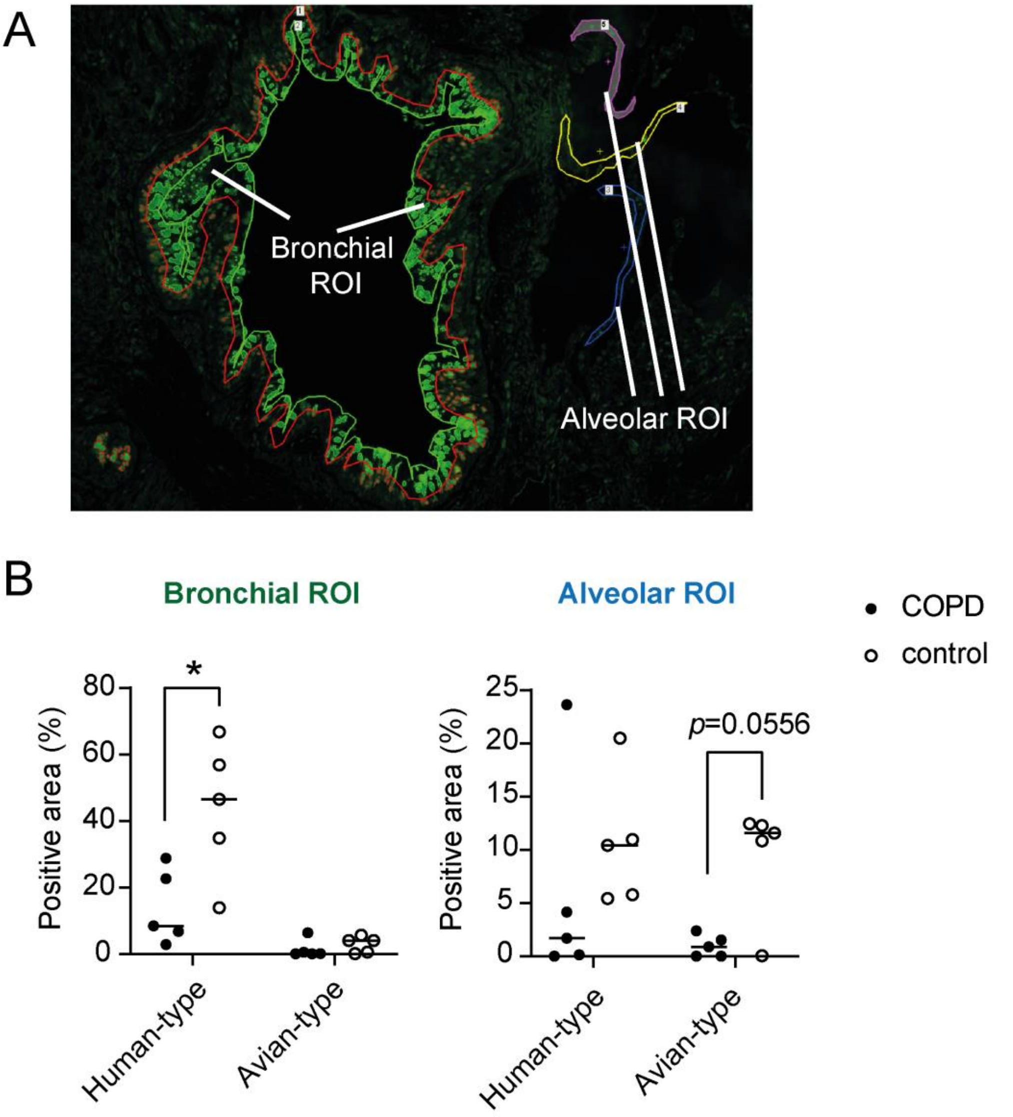

Supplementary Figure 3: TEER values of phBECs from COPD patients and controls during differentiation at the air-liquid interface. On ALI day 0, 7, 14, 21, 28 (pre-infection), TEER values were measured in every well. Three points per membrane were measured, and the average values were calculated, blank substracted, and multiplied by the membrane area (1.12 cm^2) to yield the final result in Ω x cm^2. Each plot shows the average TEER value of 28 to 36 wells measured. Vertical bars show the standard deviation. Statistical analysis was performed using an unpaired, two-sided Mann-Whitney U test, but with a cut-off of $p<0.05$ no statistically significant differences were observed.

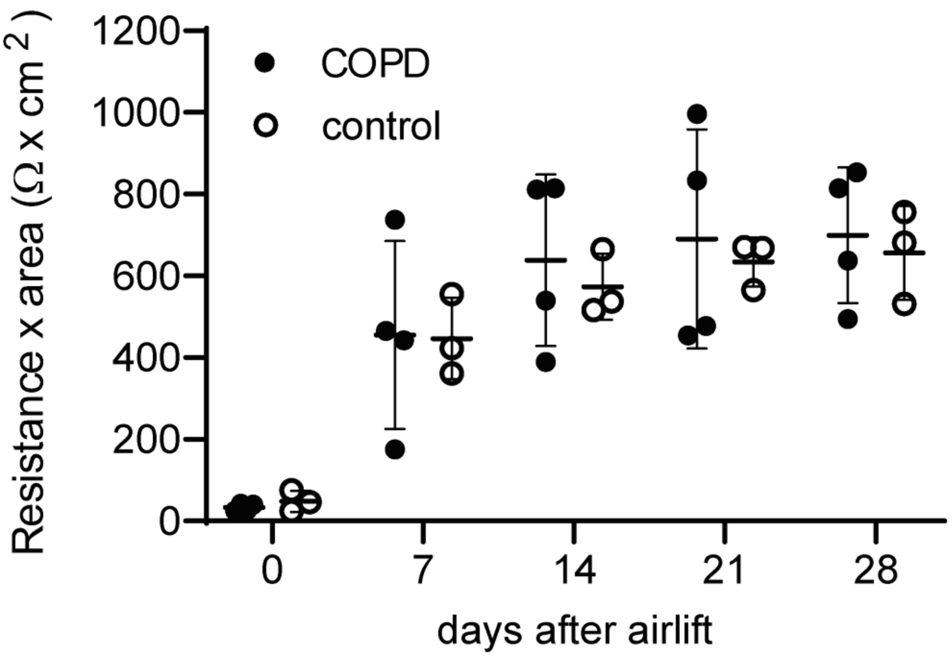

Supplementary Figure 4: Cell composition of phBECs from COPD patients and controls during differentiation at the air-liquid interface. PhBECs were cultured under the air-liquid interface for 28 days for full differentiation. Cells were collected on days 0, 14 and 28 after airlift, fixed with 4% paraformaldehyde for staining, or total RNA was extracted for qRT-PCR. (A) Expression of acTub (ciliated cells, in green), MUC5AC (goblet cells, in green) or CC10 (club cells, in green) from COPD and control on day 28 after airlift. The nucleus is stained with DAPI (blue). Representative pictures are shown. Scale bar 50 μm. (B) Quantification of the staining for acTub, MUC5AC, and CC10 on days 0, 14 and 28 after airlift. Pictures were taken from three randomly selected areas (x200) and the number of spots positive for each differentiation marker and nucleus was counted with spot detection analysis mode in Imaris (Oxford Instruments plc, UK, Abingdon). The percentage of spots detected for each differentiation marker/DAPI was calculated and average of three areas is shown as a plot for that subject. (C) qRT-PCR of Forkhead box protein J1 (FOXJ1, transcription factor specifically required for motile cilia formation), MUC5AC and CC10. Two different house keeping genes, UBC and DHX8, were used. (B) and (C) Horizontal lines show the mean, with standard deviation in vertical bars. Statistical analysis was performed using an unpaired, two-sided Mann-Whitney U test, but with a cut-off of $p<0.05$ no statistically significant differences were observed.

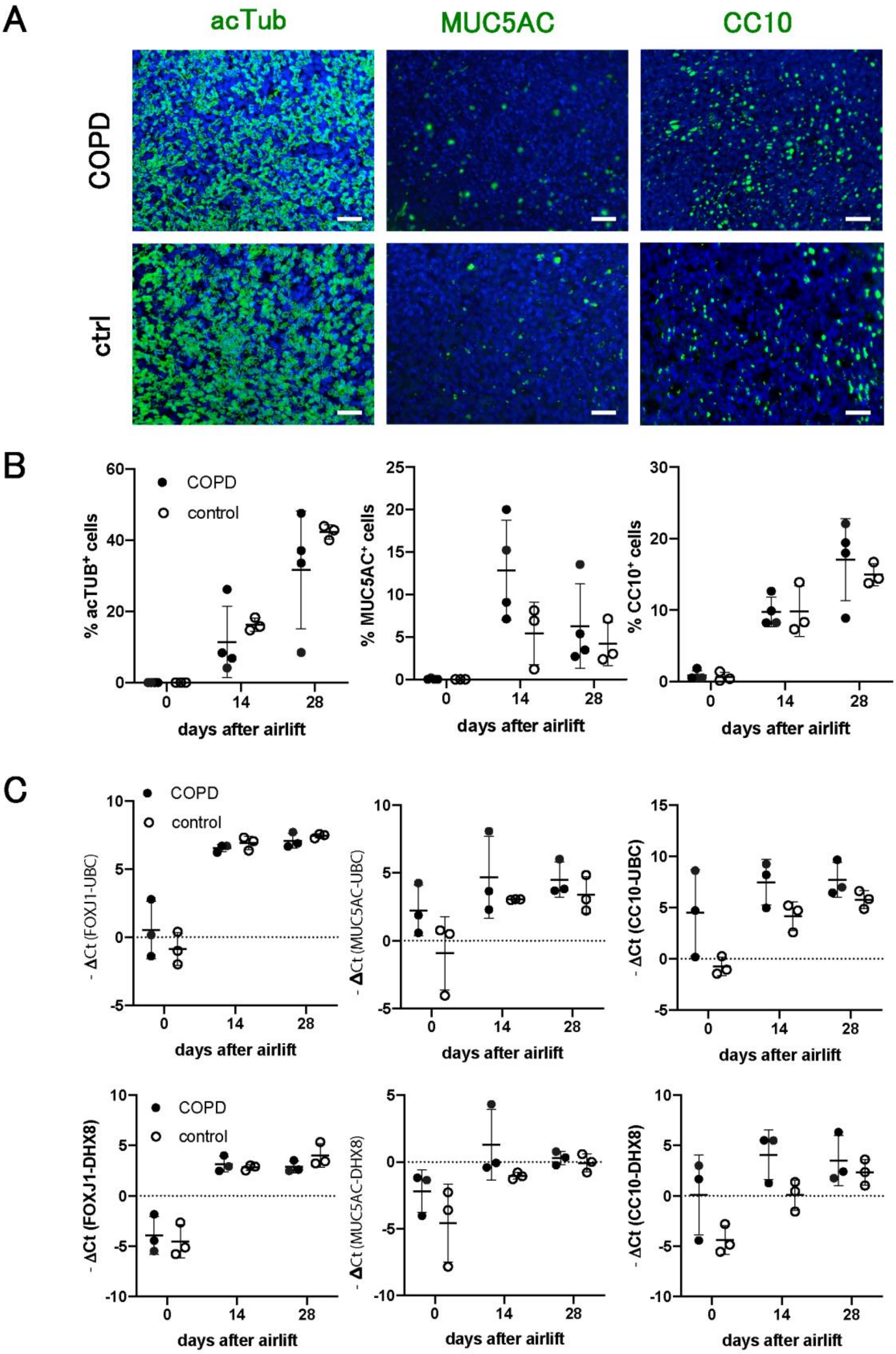
A
acTub
MUC5AC
CC10
COPD
ctrl
B
COPD
control
% acTUB⁺ cells
% MUC5AC⁺ cells
% CC10⁺ cells
days after airlift
C
- ΔCt (FOXJ1-UBC)
- ΔCt (MUC5AC-UBC)
- ΔCt (CC10-UBC)
- ΔCt (FOXJ1-DHX8)
- ΔCt (MUC5AC-DHX8)
- ΔCt (CC10-DHX8)

Supplementary Figure 5: COPD GOLD stage IV-derived phBECs (n=1) overall show more COPD-like airway features. (**A**) Data as given in Supplementary Figure S3, highlighting the COPD GOLD stage IV-derived cells in red: COPD GOLD stage IV-derived cells are characterized by a comparably lower epithelial barrier integrity. (**B**) Data as given in Figure 1C, highlighting the COPD GOLD stage IV-derived cells in red: COPD GOLD stage IV-derived cells display less human-type (but more avian-type) flu receptor. (**C**) Data as given in Supplementary Figure S4B, highlighting the COPD GOLD stage IV-derived cells in red: After 28 days of differentiation, COPD GOLD stage IV-derived cells contain fewer ciliated cells and more goblet cells. (**D**) COPD GOLD stage IV-derived phBECs show a COPD-like protein profile. Markers for COPD-derived airway epithelial cells in fully differentiated phBECs from COPD patients and controls. Typical COPD genes in bronchial airway epithelium (Steiling et al., 2013;Wei et al., 2015) are shown on the x-axis. Relative protein abundance of fully differentiated phBECs from COPD patients and controls in this study was plotted on the y-axis. (**E**) Data as given in Figure 2E/F, highlighting COPD GOLD stage IV-derived cells in red: Quantification of RNA encoding the viral nucleoprotein and viral titers indicate that PR8 virus still replicates similarly, if not even somewhat more efficiently (day 1) in COPD GOLD stage IV-derived cells.

CAMK1D, calcium/calmodulin-dependent protein kinase type 1D; CEACAM5, carcinoembryonic antigen-related cell adhesion molecule 5; CES1, carboxylesterase 1; CFB, complement factor B; DSC3, desmocollin-3; FGFBP1, fibroblast growth factor-binding protein 1; FUT3, fucosyltransferase 3; IFI44L, interferon-induced protein 44-like; IGF2BP3, insulin-like growth factor 2 mRNA-binding protein 3; INDO, indoleamine 2,3-dioxygenase 1; LCN2, lipocalin-2; MUC13, mucin-13; MUC5AC, mucin-5AC; MUC5B, mucin-5B; PSMB9, proteasome subunit beta type-9; SERPINB13, serpin B13; TCN1, transcobalamin-1; TGFB1, transforming growth factor beta-1; TNC, tenascin.

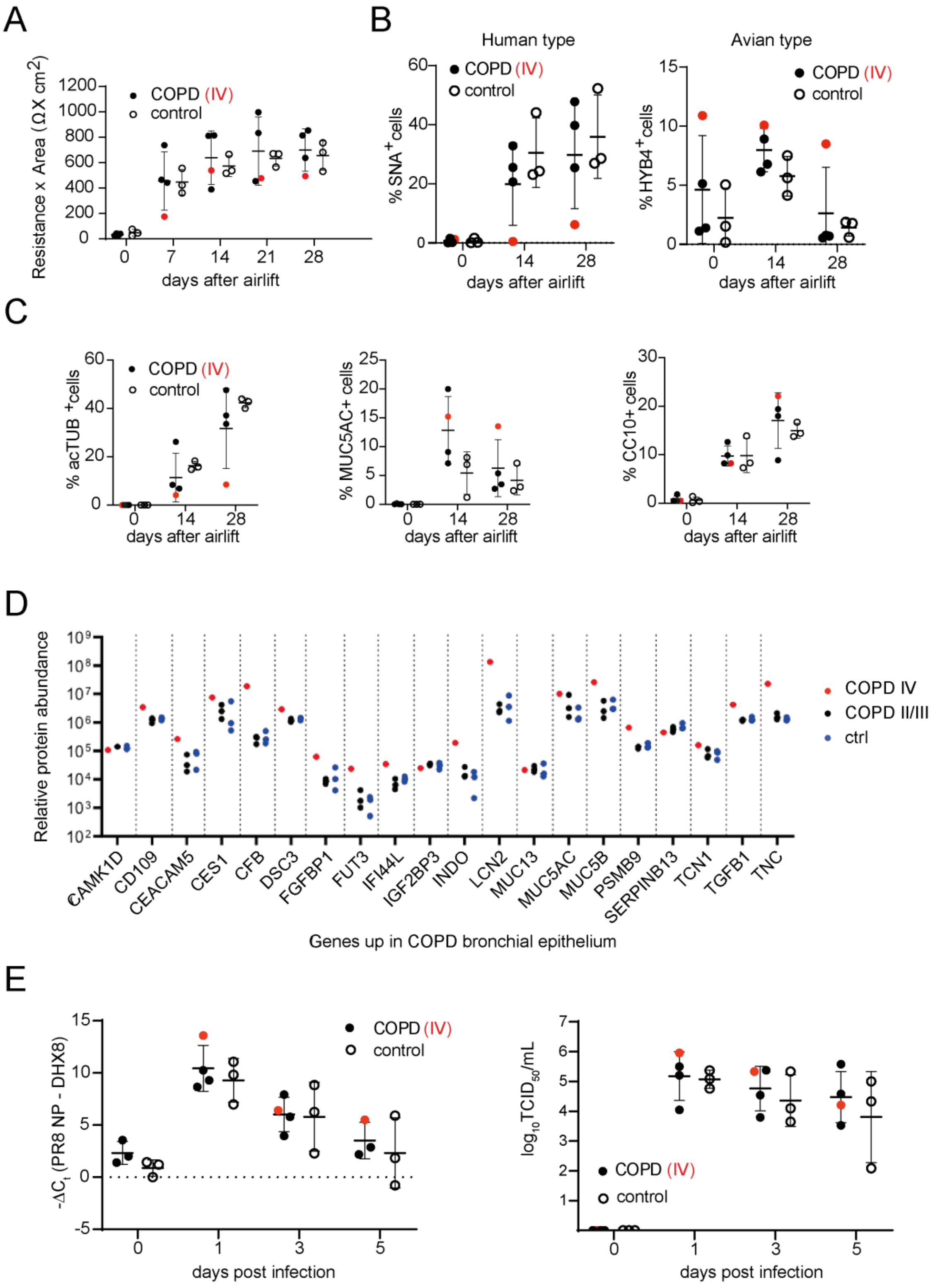

A
Resistance x Area (Ω X cm2)
COPD (IV)
control
days after airlift
B
Human type
% SNA+cells
Avian type
% HYB4+cells
COPD (IV)
control
days after airlift
C
% acTUB +cells
% MUC5AC+ cells
% CC10+ cells
COPD (IV)
control
days after airlift
D
Relative protein abundance
COPD IV
COPD II/III
ctrl
CAMK1D
CD109
CEACAM5
CES1
CFB
DSC3
FGFBP1
FUT3
IFI44L
IGF2BP3
INDO
LCN2
MUC13
MUC5AC
MUC5B
PSMB9
SERPINB13
TCN1
TGFB1
TNC
Genes up in COPD bronchial epithelium
E
-ΔCt (PR8 NP - DHX8)
log10 TCID50/mL
COPD (IV)
control
days post infection

Supplementary Figure 6: t-Distributed Stochastic Neighbor Embedding (tSNE) plots of fully differentiated phBECs post-infection. Fully differentiated phBECs were infected with influenza A virus PR8. On days 1 and 3 post-infection, cells were collected for proteomic analysis. Separation was seen in condition effect (PR8 *vs*. mock), but not in time (day 1 *vs*. day 3) or disease state (COPD *vs*. ctrl). Data were corrected for the patient effect.

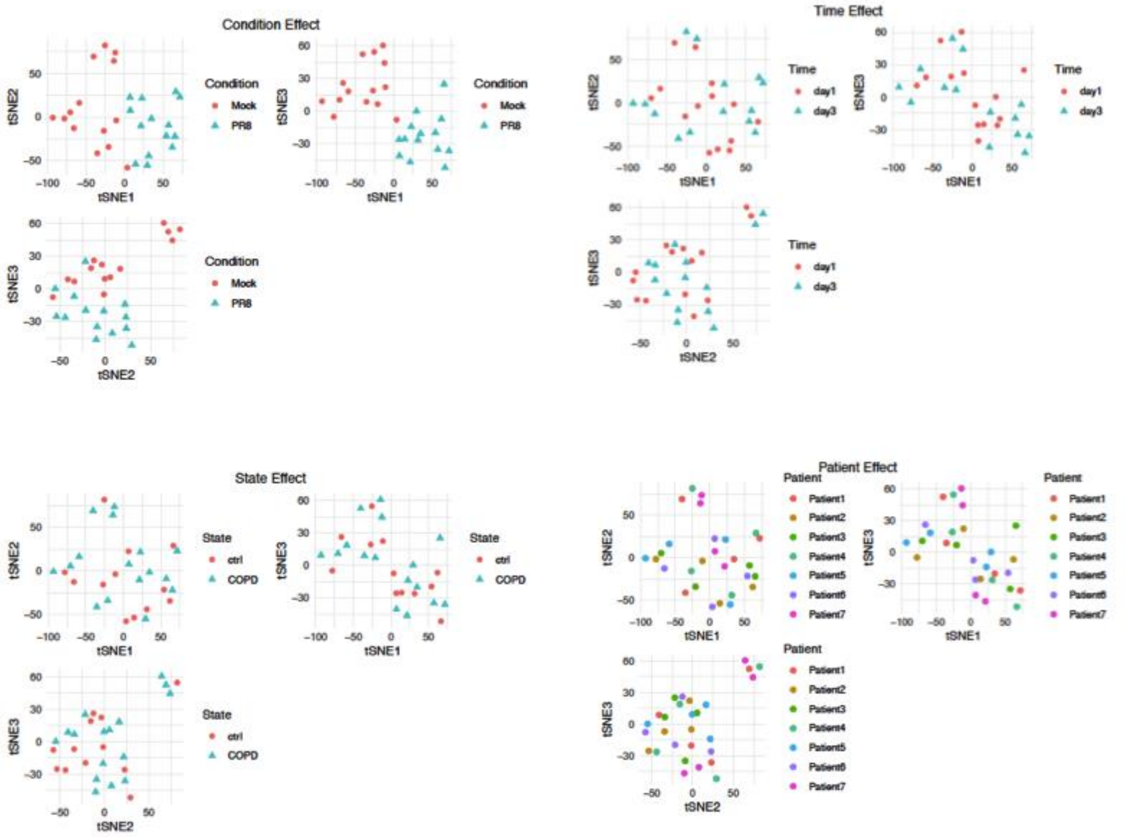

Supplementary Figure 7: Heatmap of significantly altered proteins independent of disease state 1 day post-infection. Fully differentiated phBECs were infected with influenza A virus PR8. On day 1 post-infection, cells were collected for proteomic analysis. A protein was considered to be differentially expressed if the comparison resulted in a false discovery rate (FDR) less than 5% with the Benjamini-Hochberg (BH) correction to correct multiple testing.

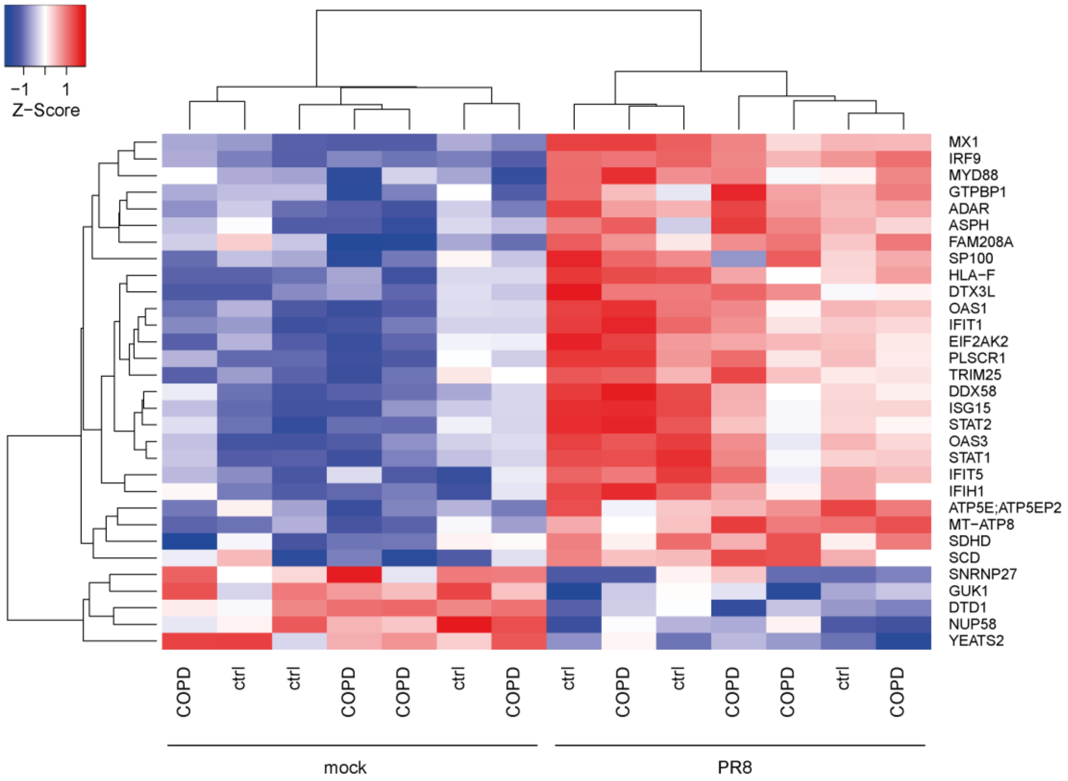

Supplementary Figure 8: Heatmap of significantly altered proteins independent of disease state 3 days post-infection. Fully differentiated phBECs were infected with influenza A virus PR8. On day 3 post-infection, cells were collected for proteomic analysis. A protein was considered to be differentially expressed if the comparison resulted in a false discovery rate (FDR) less than 5% with the Benjamini-Hochberg (BH) correction to correct multiple testing.

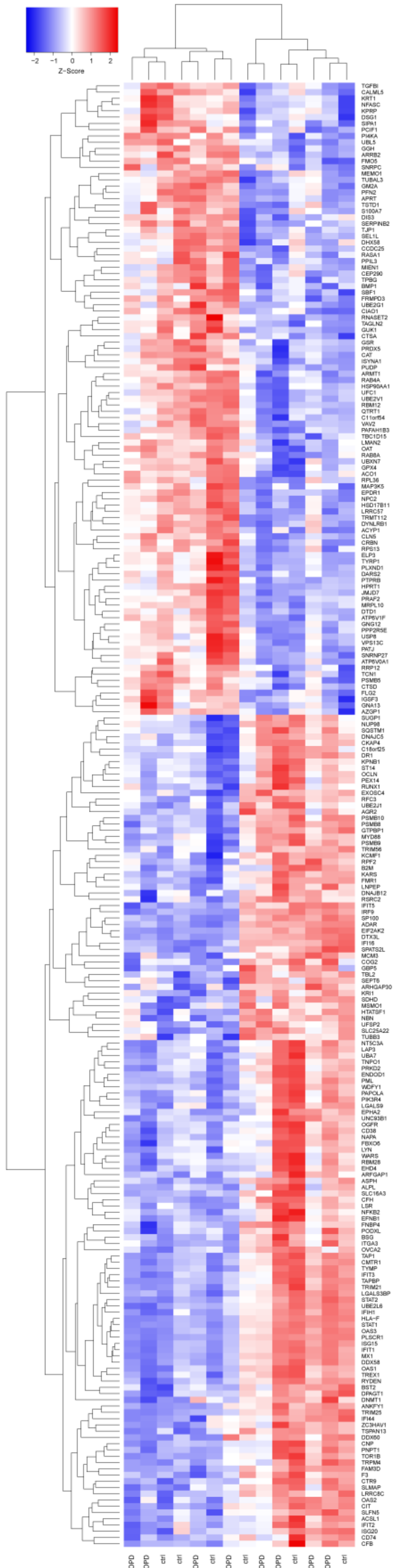

−2 −1 0 1 2
Z−Score
COPD COPD ctrl ctrl COPD ctrl COPD ctrl COPD COPD ctrl COPD COPD ctrl
mock
PR8

Supplementary Figure 9: Uncropped full-length blots to Figure 5C. Signals were documented with the ChemiDoc XRS+ Imaging System and the merged image (luminescence + photograph showing the molecular weight marker) is displayed. Protein load was low, therefore the membrane encompassing molecular weights from 35 to 250 kDa was reprobed with different antibodies. Antibodies against DTX3L and HLA-F were both from rabbit. As DTX3L was developed first and the blot was not stripped in efforts not to lose overall signal intensity, this band (upper band between 70 and 100 kDa) still is visible in the HLA-F blot. According to Uniprot (P30511), HLA-F has three isoforms ranging in size from 28 to 50 kDa, which is why we show this range in the main figure. MW, molecular weight.

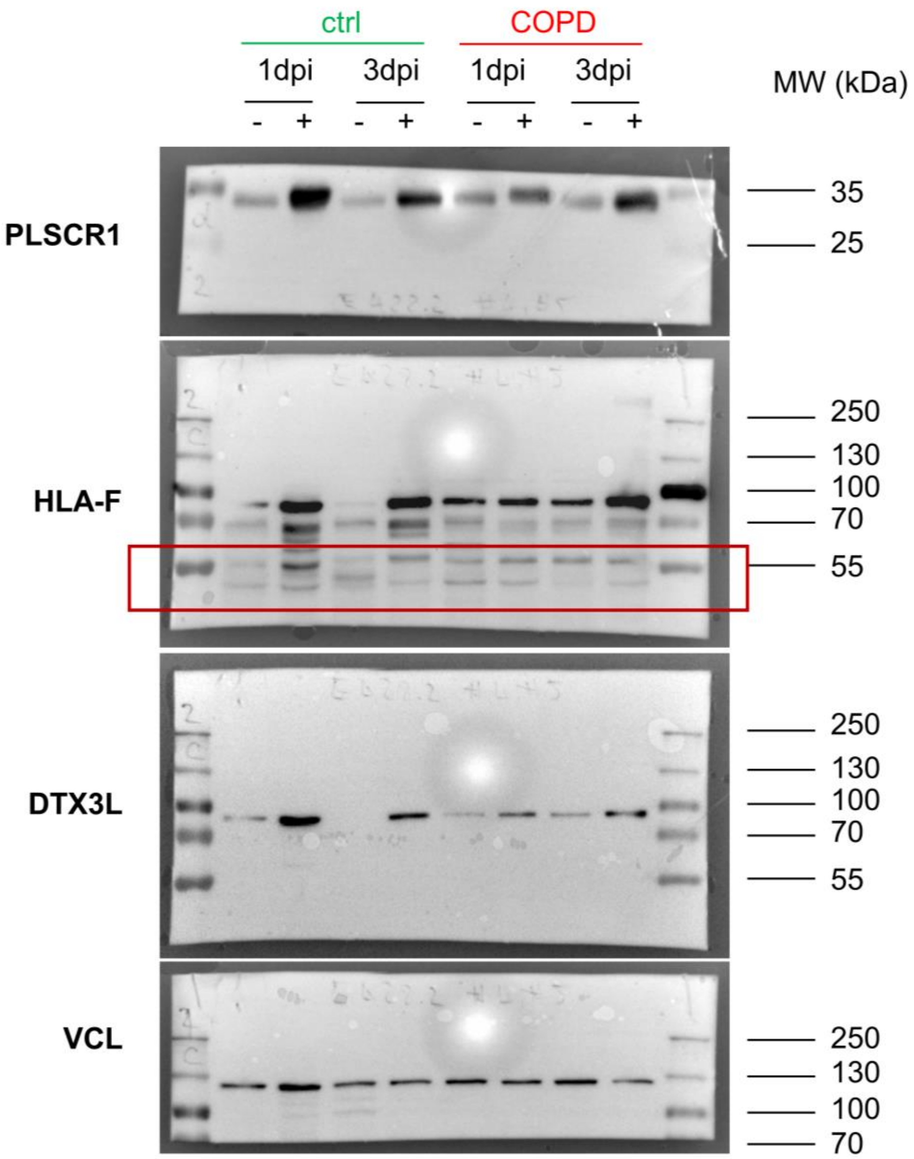

SUPPLEMENTARY TABLES

Supplementary Table 1: Patient medication.

	GOLD Stage	Primary Diagnosis	Medication (active ingredients)
COPD	IV	COPD	Torasemide, Atorvastatin, Salbutamol when required, Budesonide, Aclidinium bromide, Formoterol, Phenprocoumon, Sertraline, Mirtazapine, Cholecalciferol
COPD	III	Squamous cell carcinoma	Hydrochlorothiazide, Torasemide, Ramipril, Carbimazole, Amiodarone, Ibuprofen, Potassium citrate, Tramadol, Pantoprazole, Magnesium citrate/L-glutamate, Sodium perchlorate, Atenolol, Tinzaparin, Tiotropium bromide
COPD	II	Squamous cell carcinoma	Nebivolol, Ezetimib, Simvastatin, Acetylsalicylic acid, Ibuprofen when required
COPD	II	Carcinoma	Irbesartan, Hydrochlorothiazide, Omeprazole, Simvastatin, Allopurinol, Tiotropium bromide
Control	NA	Primary histiocytic sarcoma	Pantoprazole, Zopiclon, Rivaroxaban
Control	NA	Pleiomorphic carcinoma	no long-term medication recorded
Control	NA	Adenocarcinoma	no long-term medication recorded

Supplementary Table 2: Primary antibodies used for immunohistochemistry/ Western blot (WB)

Target	Origin and Clone	Company	Dilution
acTub	Rabbit, EPR16772	ab179484, Abcam PLC, Cambridge, UK	1:500 (phBEC)<br>1:1000 (FFPE lung)
MUC5AC	Mouse, 45M1	ab3649, Abcam	1:250 (phBEC and FFPE lung)
CC10	Rabbit polyclonal	sc25554, Santa Cruz Biotechnology, Inc, TX	1:300 (phBEC)<br>1:100 (FFPE lung)
CK5	Rabbit, EP1601Y	Ab52635, Abcam	1:200 (FFPE lung)
Avian type-flu receptor<br>(Sia α2,3)	Mouse, HYB4	011-25171, FUJIFILM Wako Chemicals, Osaka, Japan	1:100 (phBEC)<br>1:20 (FFPE lung)
PLSCR1	Rabbit polyclonal	11582-1-AP, Proteintech Group, Inc, IL	1:1000 (WB)
HLA-F	Rabbit polyclonal	AV49411-100UL, Sigma-Aldrich CO LLC, MO	1:1000 (WB)
DTX3L	Rabbit polyclonal	11963-1-AP, Proteintech Group, Inc	1:1000 (WB)
Vinculin	Mouse monoclonal	V9131, Sigma-Aldrich	1:1000 (WB)

Supplementary Table 3: Secondary antibodies used for immunohistochemistry/ Western blot

Target	Label	Company	Dilution
Rabbit IgG	Alexa Fluor 488	ThermoFisher Scientific, A-11008	1:400
Mouse IgG	Alexa Fluor 568	Alexa Fluor 568 goat anti-mouse IgG (H+L), Invitrogen, A11004	1:400
Rabbit IgG	Alexa Fluor 568	Alexa Fluor 568 goat anti-rabbit IgG (H+L), Invitrogen, A11011	1:400
Enhanced chemiluminescence Anti-Rabbit IgG	Horse raddish peroxidase	GE Healthcare UK limited, NA934	1:60000
Enhanced chemiluminescence Anti-Mouse IgG	Horse raddish peroxidase	GE Healthcare UK limited, NA931	1:60000

Supplementary Table 4: Primers used for qRT-PCR

Target	Forward primer (5'>3')	Reverse primer (5'>3')
human FOXJ1	TCGTATGCCACGCTCATCTG	CTTGTAGATGGCCGACAGGG
human MUC5AC	AGCAGGGTCCTCATGAAGGTGGAT	AATGAGGACCCCAGACTGGCTGAA
human CC10	TTCAGCGTGTCATCGAAACCC	ACAGTGAGCTTTGGGCTATTTTT
human PLSCR1	ACTGTTTTAATCATGGACAA	GGATACTGAGGAGGATACC
human HLA-F	GCACAGACTGACCGAGT	TTCCCTGGAGGGTGT
human CMTR1	CAAGGACTCTACTTTTGACC	AGTCATGCACACGAATG
human DTX3L	AAAGAAGAACACGAGACCT	GTCAGCTGTTACAGTAAGAAAG
human SHFL	AGTTTCATGGGAAGGTATC	TACTTAGTTCTTGGCCATCT
human DHX8	TGACCCAGAGAAGTGGGAGA	ATCTCAAGGTCCTCATCTTCTTCA
human ubiquitin	CACTTGGTCCTGCGCTTG	TTTTTTGGGAATGCAACAACTT
PR8 nucleoprotein	CCCAGGATGTGCTCTCTGAT	TTCGTCCATTCTCACCCCTC

Supplementary references

Steiling, K., Van Den Berge, M., Hijazi, K., Florido, R., Campbell, J., Liu, G., Xiao, J., Zhang, X., Duclos, G., Drizik, E., Si, H., Perdomo, C., Dumont, C., Coxson, H.O., Alekseyev, Y.O., Sin, D., Pare, P., Hogg, J.C., Mcwilliams, A., Hiemstra, P.S., Sterk, P.J., Timens, W., Chang, J.T., Sebastiani, P., O'connor, G.T., Bild, A.H., Postma, D.S., Lam, S., Spira, A., and Lenburg, M.E. (2013). A dynamic bronchial airway gene expression signature of chronic obstructive pulmonary disease and lung function impairment. *American Journal of Respiratory and Critical Care Medicine* 187**,** 933-942.

Wei, L., Xu, D., Qian, Y., Huang, G., Ma, W., Liu, F., Shen, Y., Wang, Z., Li, L., Zhang, S., and Chen, Y. (2015). Comprehensive analysis of gene-expression profile in chronic obstructive pulmonary disease. *International Journal of Chronic Obstructive Pulmonary Disease* 10**,** 1103-1109.
